# The class III peroxidase gene family is involved in ascorbic acid induced delay of internal browning in pineapple

**DOI:** 10.3389/fpls.2022.953623

**Published:** 2022-08-03

**Authors:** Xiaowan Hou, Zhiwei Lu, Keqian Hong, Kanghua Song, Hui Gu, Wei Hu, Quansheng Yao

**Affiliations:** ^1^Key Laboratory for Postharvest Physiology and Technology of Tropical Horticultural Products of Hainan Province, South Subtropical Crop Research Institute, Chinese Academy of Tropical Agricultural Sciences, Zhanjiang, China; ^2^Biotechnology Research Institute, Chinese Academy of Agricultural Sciences, Beijing, China; ^3^Key Laboratory of Forage and Endemic Crop Biotechnology, Ministry of Education, School of Life Sciences, Inner Mongolia University, Hohhot, China; ^4^Key Laboratory of Biology and Genetic Resources of Tropical Crops, Institute of Tropical Bioscience and Biotechnology, Chinese Academy of Tropical Agricultural Sciences, Haikou, China

**Keywords:** *Ananas comosus*, internal browning, ascorbic acid, the class III peroxidase, reactive oxygen species

## Abstract

Excessive production of reactive oxygen species (ROS) leads to potential toxicity in an organism. Class III peroxidases (PRXs) play an important role in maintaining ROS homeostasis in plants. Internal browning (IB) limits industrial development of pineapple, which is the third most important fruit trade in the world. IB is mainly caused by ROS, and the mechanism underlying IB is still unknown from the perspective of ROS. Here, we soaked pineapples in ascorbic acid after harvest and before storage to decrease excessive ROS and polyphenol oxidase (PPO) activity, ultimately restraining the spread and deterioration of IB. Using phylogenetic analysis; we identified 78 pineapple PRX genes (*AcPRXs*) and divided them into five subgroups. Gene structure analysis indicated that the exon numbers ranged from 2 to 14, and conserved motif analysis verified that all of the *AcPRXs* identified here have standard peroxidase domains. Analysis of duplication events suggested that tandem and segmental duplication events may have played equal and important roles in expanding the AcPRX family. Comprehensive transcriptomic analysis uncovered that *AcPRXs* may play an important role in negatively regulating the occurrence of IB. In summary, we found that ROS scavenging delayed IB occurrence. The results of characterized AcPRX family revealed that AcPRXs family responded to growth and development, and negatively regulated to IB occurrence in storage stage. This research provides potential target genes for future in-depth analysis of the molecular mechanisms underlying IB and contributes to develop IB-resistant pineapple varieties.

## Introduction

Pineapple (*Ananas comosus* [L.] Merr) is the third most important fruit trade in the world after bananas and mangoes and has important industrial and nutritional value in tropical and subtropical countries. Internal browning (IB) is a physiological disorder in pineapple during the postharvest storage stage, and it causes heavy damages to fruit quality and the long-term development of the pineapple industry (Zhang et al., 2016). The previous researches suggested that polyphenol oxidase (PPO), phenolic substrate, and excessive reactive oxygen species (ROS) were three necessary factors leading to its occurrence (Hassan et al., 2010; Kietsuda Luengwilaia, 2016). When pineapples are continuously exposed to low temperatures (either in the field or during postharvest storage), ROS bursts occur, lead to membrane damage (Zhou et al., 2014). As a result, plastid PPOs and vacuole-localized polyphenols are released from their respective organelles, allowing polyphenols to be oxidized into o-quinones and leading to the occurrence of IB (Zhou et al., 2003a; Raimbault et al., 2011; Hu et al., 2012; Kietsuda Luengwilaia, 2016). So far, most of the reports about the mechanisms underlying IB occurrence focus on the functions of AcPPOs and phenolics formation, but the effects of ROS have received comparatively little attention.

Reactive oxygen species generation in plants is a common phenomenon under both normal and stress environments (Sachdev et al., 2021). ROS play important roles in controlling various biological processes, including plant growth, development, and biotic and abiotic stresses in complex environments (Choudhury et al., 2017; Tao et al., 2018). Nonetheless, ROS is a double-edged sword. When biotic or abiotic stresses become further more serious, ROS gradually accumulate. Once the rate of ROS accumulation exceeds the capacity of ROS scavenging, ROS homeostasis is broken. This is accompanied by potential toxicity, membrane damage, and DNA injuries, finally, plants may undergo early flowering or programmed cell death (Vanderauwera et al., 2011; Petrov et al., 2015; Sewelam et al., 2016; Noctor Graham, 2017; Hasanuzzaman et al., 2019). Therefore, maintenance of ROS homeostasis *in vivo* is necessary for normal regulation of growth, development, and stress responses (Chaves, 2011; Qu et al., 2013).

To better adapt to the constantly-changing external environment, plants have evolved a set of antioxidant defence systems including peroxidases, NADPH oxidases (NOXs), and antioxidants to scavenge excessive ROS *in vivo* (Camejo et al., 2016; Hasanuzzaman et al., 2019; Mbadinga et al., 2020). Peroxidases, the primary component of the antioxidant defence system, are haem-containing oxidases that employ hydrogen peroxide as the electron acceptor to catalyse a series of oxidative reactions (Almagro et al., 2009; Mbadinga et al., 2020). The reduction of H_2_O_2_ and the formation of ROS are mainly achieved by shuttling electrons to various donor molecules, such as phenolic compounds, lignin precursors, auxin, or secondary metabolites (Mathé et al., 2010; Camejo et al., 2016; Wu et al., 2019). Depending on the species in which they are found, peroxidases are divided into two superfamilies: one found in bacteria, fungi, and plants, and the second found in animals. The first superfamily has three major classes: intracellular (class I), fungal-secreted (class II), and plant-specific secreted (class III; Welinder, 1992; Mathé et al., 2010; Li et al., 2020b). Class III peroxidases are recognized as the main enzymes responsible for generation and scavenging of ROS, and are abbreviated as peroxidase (PRX), POD, POX, and PER (Almagro et al., 2009; Wu et al., 2019). Research has shown that genes belonging to class III peroxidases have roles in maintaining ROS homeostasis and alleviating oxidative damage to cope with diverse stresses such as high temperature (Kim et al., 2011), salt (Kim et al., 2013), drought (Zhang et al., 2019; Su et al., 2020), heavy metal exposure (Maria Kidwai et al., 2019), and disease (Wally and Punja, 2010).

It was previously reported that IB could be delayed or minimized by reducing phenolic biosynthesis and inhibiting PPO activity (Zhou et al., 2003b; Zhang et al., 2015). Despite these advances, the molecular mechanisms underlying IB remain ill-defined, limiting development of effective strategies for preventing IB. However, as an important factor that can trigger IB occurrence, ROS homeostasis is an ideal entrance point for clarifying the molecular mechanisms underlying IB, which is maintained by members of the peroxidase family and antioxidants. Ascorbic acid (AsA), a direct ROS scavenger and antioxidant, can restrain PPO activity and increase total antioxidant activity (Altunkaya and Gokmen, 2008). Yun et al. (2021) showed that reductions in AsA increased the speed of peel browning and pulp decay in litchi. In the present study, pineapples were treated with 0.2% AsA to illustrate the effects of exogenous AsA treatment on IB characteristics in pineapple. To further explore whether peroxidases participate in regulation of IB in pineapple, evolutionary relationships and gene expression of the pineapple class III PRX gene family were analysed. This study provides a new strategy for pineapple preservation and a novel perspective for studying the mechanism of IB occurrence.

## Materials and methods

### Plant materials and treatments

**Figure 1:**
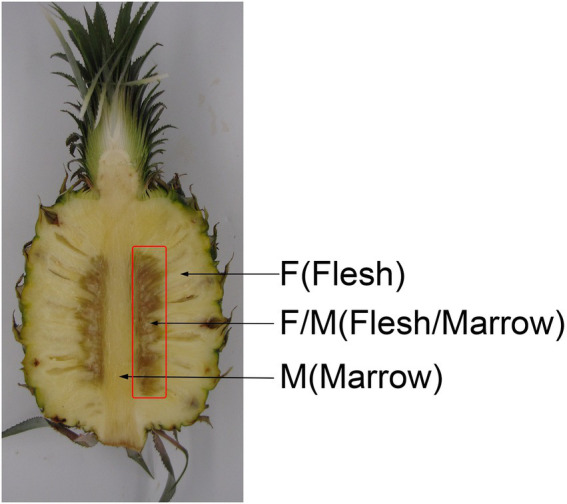
Bisected pineapple showing the area sampled.

Pineapple fruits (*A. comosus* cv. Comtede Paris) grown in Xuwen County, Guangdong Province, China were harvested at 60% maturity (partial flat eyes and nearly 100% green area, equating to commercial maturity in China). Fruit bottoms were soaked into 0.05% (w/v) metiamide for 1 min to prevent fungal disease. After air drying, fruits with the following traits were selected: no visual evidence of pests or disease; no mechanical damage; similar fruit size; and weighing 0.9 ± 0.05 kg. A total of 240 pineapples were selected and were equally divided at random into two groups. Fruits were treated for 15 min with either a water control or 0.2% (m/v) AsA. The concentration of AsA used was determined from preliminary experiments using 0, 0.1, 0.2, 0.4, and 0.5% AsA. After air drying outdoors for 3 h, fruits were stored in a fruit storehouse at 25 ± 2°C with 85–95% humidity. Treated pineapples were sampled at three timepoints: translucency symptoms (day 3), tissue browning (day 6), and tissue browning spreading into the flesh or marrow (day 9). Samples were taken from the flesh/marrow (F/M; Figure 1 referenced Kietsuda Luengwilaia, 2016), frozen in liquid nitrogen, and stored at −80°C. At each sampling point, 30 fruits were used for record IB incidence and taking samples. There were three biological replications, each containing 30 pineapples.

### Assessment of IB incidence and IB index

Internal browning incidence was calculated as the proportion of fruits with IB spots. IB index was defined using five levels as described by Gong (2010). Level 0 meant there were no dark spots or discoloration; level 1 meant dark spots were beginning to appear, but occupied a cross-sectional area of less than 10%; level 2 meant dark spots occupied a cross-sectional area of 11–20%; level 3 meant dark spots occupied a cross-sectional area of 21–30%; level 4 meant dark spots occupied a cross-sectional area of 31–50%; and level 5 meant dark spots occupied a cross-sectional area of more than 50%. The formula for calculating IB index is as follows:


IBindex=[∑(SiNi)/5N]×100

where *S* represents the disease grade, *i* represents the number of levels at each grade, *N*i represents the number of diseased fruits at the corresponding level, and *N* represents the total number of fruits investigated. The pineapple fruits in each treatment group were halved lengthwise, and IB incidence and IB index were observed and recorded.

### IB and ROS marker and enzyme activity measurements

Pineapple fruits were smashed with a pulverizer, and then stored in liquid nitrogen prior to measuring malondialdehyde (MDA), H_2_O_2_, AsA, and PPO and PRX enzyme activity. MDA was measured using the thiobarbituric acid (TBA) method. H_2_O_2_ content was determined by calculating the quantity of ammonium molybdate at 405 nm based on the reaction between H_2_O_2_ and molybdic acid. Endogenous AsA content was measured by 2, 6-dichlorophenol indophenol titration (El Bulk et al., 1997). PPO activity was assayed by adding catechol then measuring quinone (which has characteristic absorbance values at 525 nm) as described by Lin et al. (2011); the same methods were applied to extract and assay the activity of PRX. All experiments were performed with three biological replicates.

### Identification of class III peroxidase gene family members in pineapple

Genome data for pineapple were obtained from the Pineapple Genomics Database (PGD; http://plants.ensembl.org/Ananas_comosus/Info/Index) as previously described (Xu et al., 2018). The full-length amino acid sequences of all rice and Arabidopsis class III peroxidase proteins were obtained from RAP^1^ and TAIR,^2^ respectively, and used as reference sequences to identify homologs in pineapple *via* BLASTP. An *E*-value of 1e^−5^ and >50% identity were used as the thresholds. The PGD database was also searched using the phrase “III peroxidase.” After wiping off redundant sequences, the final candidate PRX protein sequences were submitted to SMART^3^ and CDD^4^ to verify the presence of the conserved PRX domains. The verified pineapple PRX genes (*AcPRXs*) were aligned with ClustalX (2.0) using the default parameters (Larkin et al., 2007).

### Phylogenetic analysis and classification of AcPRX genes

Phylogenetic analysis was conducted with the full-length amino acid sequences of verified AcPRXs (Supplementary File 1). First, all of the AcPRX sequences were aligned with ClustalX (v1.83; Thompson et al., 1997) using the default parameters. MEGA6 was then used to construct an unrooted neighbour-joining phylogenetic tree with a bootstrap value of 1,000 (Tamura et al., 2013). Finally, AcPRX genes were divided into different subgroups based on tree topology.

### Sequence analysis and structural characterization

Pineapple PRX genes amino sequences were submitted to ExPASy^5^ to determine the amino acid length, molecular weight, and theoretical isoelectric point (pI) of each protein. The chromosomal distributions and number of exons for each gene were obtained from the PGD. Conserved motifs in the AcPRX sequences were identified with MEME v5.1.1^6^ using the following parameters: any number of repetitions, 10 motifs, and an optimum motif width of 10–50 amino acid residues. Conserved motifs were further annotated by the InterPro online database.^7^ The Gene Structure Display Server online^8^ was used to identify the exon-intron structures (Hu et al., 2015).

### Chromosomal localization and gene duplication

The chromosomal distributions and relative distances of *AcPRXs* were mapped using MapChart software (Voorrips, 2002). Pairs of AcPRXs were determined to be derived from gene duplication if the following two criteria were met: (1) the similarity between the longer sequence and the shorter sequence was >70%, and (2) the shorter sequence length was >70% of the longer sequence length (Gu et al., 2002; Yang et al., 2008). Two genes that were separated by five or fewer genes in a 100-kb chromosome fragment were regarded as tandem duplicates (Wang et al., 2010) The PGD has records of segmental duplication events, which were searched to designate AcPRXs as segmental duplicates where appropriate. Ka (Non-synonymous) and ks (synonymous; ks) of each duplicated *AcPRX* genes were obtained by KaKs_Calculator 2.0. The divergence time (T) was obtained with the equation of T = Ks/(2 × 6.1 × 10^−9^) × 10^−6^ million years ago (mya).

### Expression profile analysis of the *AcPRXs* genes at different stages of IB

Using the PGD website (http://pineapple.angiosperms.org/pineapple/html/index.html; Ming et al., 2015). Expression profiles of *AcPRXs* were analysed in multiple datasets derived from different organizations and various developmental stages. *AcPRX* expression patterns were analysed according to our previous RNA-seq data, in which AsA-treated and control fruits at days 0, 4, and 6 after storage were sampled for RNA-seq analysis. The sampled method was based on the article’s “Plant materials and treatments” parts. All expression profiles were displayed as heatmaps, which were generated using TBtools (Chen et al., 2020). Quantitative real-time PCR (qRT-PCR) was performed to validate the RNA-Seq data and confirm expression patterns of *AcPRXs* in response to AsA treatment. qRT-PCR primers in Supplementary Table 4.

### Data analysis

All data handling, statistics, and plots were performed using SigmaPlot® version 10.0 Software.^9^ Data were described as mean ± SD; Student’s *t*-test and Duncan’s test were used to compare. *p* ≤ 0.05 or less was considered statistically significant.

## Results

### Effect of AsA on pineapple IB

**Figure 2:**
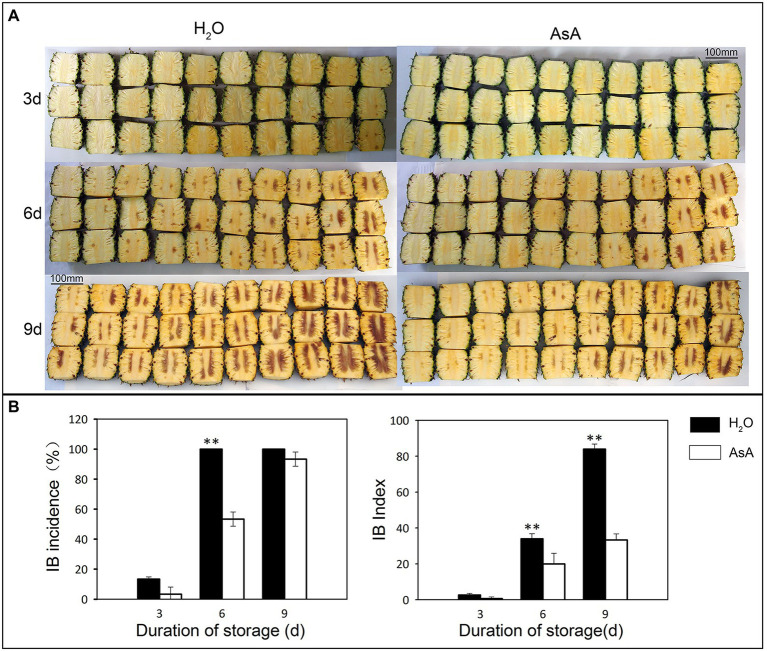
Effects of exogenous ascorbic acid treatment on internal browning (IB) in pineapples at different storage timepoints. The phenotype **(A)** IB incidence and IB index **(B)** of different treatment groups. **p ≤ 0.01 (Student’s *t*-test).

Exogenous application of AsA appeared to act as a buffer, inhibiting spread, and progression of IB in postharvest pineapple. The time to first IB occurrence was the same between control and AsA treatment group; there were translucency symptoms at 3 days after treatment, then transition to browning of the fresh tissue at 6 days (Figure 2A). The IB index and the incidence of IB showed significant differences between the groups at 6 days (*p* ≤ 0.01; Figure 2B). After AsA treatment, the occurrence of IB had dropped to 53.33% compared to 100% in the control. At 9 days, there were no differences in the incidence of IB, although the IB index was for 84 and 33.33 in the control and AsA-treated group respectively, which was significantly different (*p* ≤ 0.01). Those results showed AsA as buffer to delay the spread and progression of IB.

### Effect of AsA on contents of H_2_O_2_, AsA, and MDA, and activities of PPO and PRX

**Figure 3:**
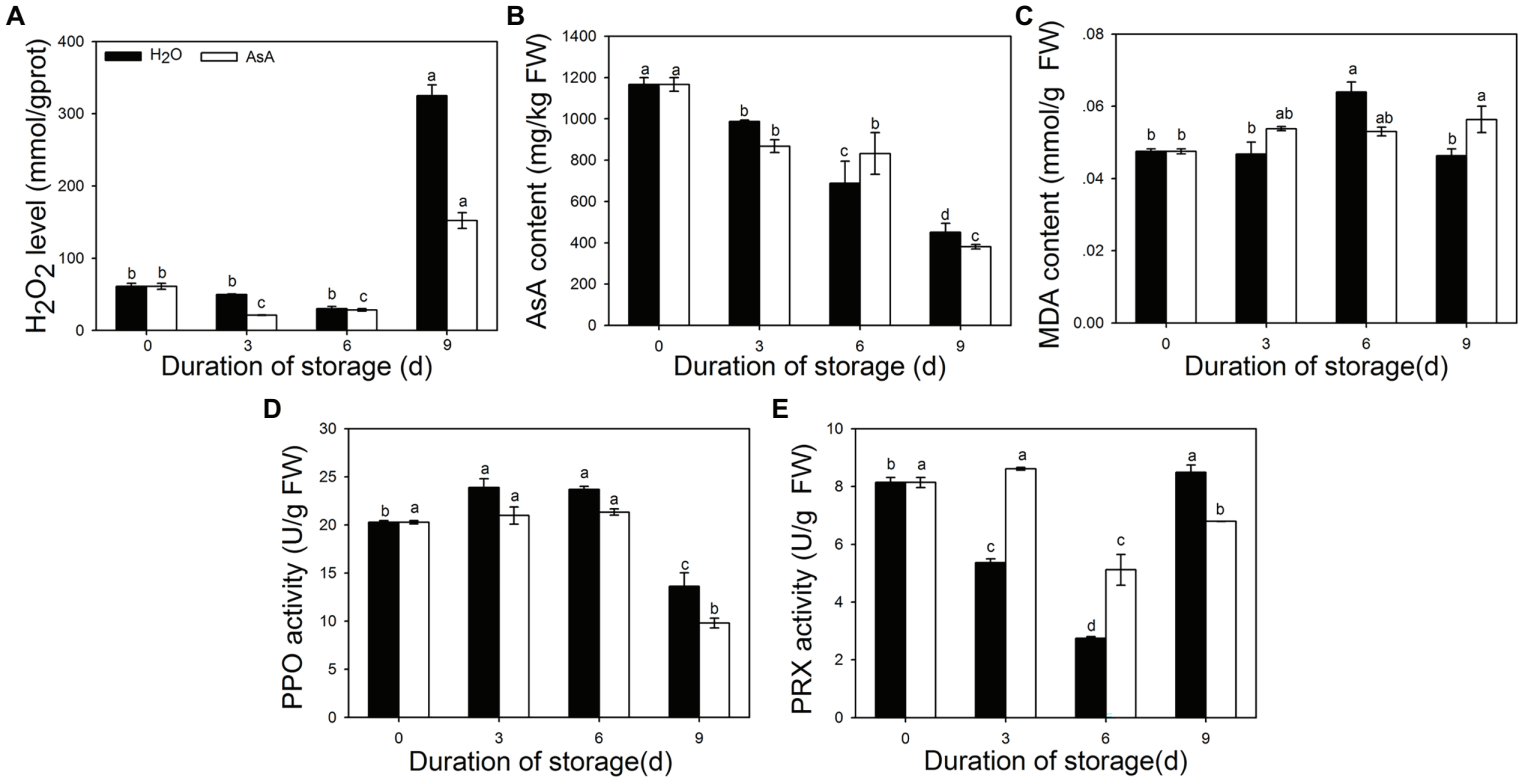
Effects of exogenous ascorbic acid treatment on several parameters in postharvest pineapple, namely H_2_O_2_ content **(A)**, Ascorbic acid (AsA) content **(B)**, malondialdehyde (MDA) content **(C)**, Polyphenol oxidase (PPO) activity **(D)**, and peroxidase (PRX) activity **(E)**. Lowercase letters indicate statistically significant differences (Duncan’s new multiple range test, *p* ≤ 0.05).

To better understand changes in pineapple as a result of AsA treatment, IB and ROS-related characteristics (such as MDA, AsA, and H_2_O_2_ levels and PPO and PRX activity) were measured at several timepoints. Fluctuations in H_2_O_2_ and AsA content (Figures 3A,B) and in PPO activity (Figure 3D) were smaller in the AsA-treated group than in the control group. Over the entire duration of storage, the maximum value of MDA content (Figure 3C) was 11.9% lower in the AsA group than the control group and delayed to occur at 9 days. In contrast, PRX activity (Figure 3E) was significantly higher at 3 and 6 days compared with the control group, showing that AsA could inhibit the accumulation of free radicals. Intriguingly, there was a consistent phenomenon among these indicators; AsA, H_2_O_2_, and MDA levels and PPO activity remained stable between 3 and 6 days in the treatment group, which likewise maintained high PRX activity during that time period.

### Genome-wide identification and analysis of *AcPRXs*

We next identified and analysed PRX genes in pineapple (*AcPRXs*). Originally, 92 *AcPRXs* were obtained by searching for homologs of known rice and Arabidopsis PRXs using BLASTP, and 105 *AcPRXs* were obtained by keyword matching in the PGD database. After eliminating the redundant sequences and verifying the presence of the conserved peroxidase domain, a total of 78 *AcPRXs* were acquired by local Hidden Markov Model analysis and renamed based on their chromosomal locations (Table 1). The lengths of the AcPRX proteins varied from 100 (AcPRX64) to 1057 (AcPRX66) amino acids, and the molecular weights ranged from 11.33 (AcPRX64) to 118.18 (AcPRX73). The predicted PI values were between 4.52 (AcPRX59) and 10.28 (AcPRX78). *AcPRX* genes were found on 22 pineapple chromosomes.

**Table 1:** Characteristics of AcPRXs confirmed in pineapple.

Name	Gene ID	Chr.	Genomic Location	ORF	Exon	AA	MW (kDa)	pI
AcPRX1	Aco006655	LG01	22,942,404–22,945,339	978	3	325	34.76	4.98
AcPRX2	Aco021354	LG01	10,118,322–10,124,972	1,059	4	352	37.51	4.82
AcPRX3	Aco021355	LG01	10,152,822–10,159,776	1,062	5	353	37.99	5.44
AcPRX4	Aco021357	LG01	10,182,978–10,185,914	993	4	330	34.94	4.61
AcPRX5	Aco022453	LG01	5,181,655–5,183,616	1,014	2	337	35.61	6.59
AcPRX6	Aco000925	LG02	15,616,393–15,618,018	1,056	4	351	38.15	4.99
AcPRX7	Aco020478	LG02	9,486,642–9,488,956	990	4	329	35.53	4.69
AcPRX8	Aco020482	LG02	9,502,096–9,508,514	993	4	330	35.88	6.59
AcPRX9	Aco011877	LG03	13,191,903–13,199,186	2094	11	697	75.87	4.81
AcPRX10	Aco014075	LG03	128,210–129,408	966	2	321	34.88	6.71
AcPRX11	Aco014076	LG03	123,997–125,071	930	2	309	33.03	5.28
AcPRX12	Aco002056	LG04	5,183,006–5,187,859	996	4	331	36.44	8.69
AcPRX13	Aco002344	LG04	2,268,585–2,275,634	1752	6	583	62	7.16
AcPRX14	Aco011124	LG04	13,559,958–13,561,759	975	3	324	34.67	4.61
AcPRX15	Aco011125	LG04	13,553,572–13,555,336	936	4	311	33.34	6.52
AcPRX16	Aco011128	LG04	13,524,180–13,526,013	963	4	320	34.36	6.8
AcPRX17	Aco014948	LG04	10,852,102–10,854,365	867	5	288	30.39	9.21
AcPRX18	Aco021983	LG04	181,855–183,224	963	4	320	34.28	9.12
AcPRX19	Aco021984	LG04	190,356–192,628	993	2	330	36.14	9.28
AcPRX20	Aco023522	LG04	374,033–376,168	993	2	330	36.09	9.27
AcPRX21	Aco023523	LG04	381,622–383,417	987	3	328	35.15	9.18
AcPRX22	Aco004317	LG05	2,129,882–2,143,830	3,060	10	1,019	109.73	8.74
AcPRX23	Aco004613	LG05	4,458,890–4,468,179	1,467	7	488	52.01	6.06
AcPRX24	Aco004784	LG05	5,778,917–5,781,088	960	4	319	34.87	5.36
AcPRX25	Aco002775	LG06	11,491,541–11,493,377	1,068	3	355	39.02	5.2
AcPRX26	Aco002920	LG06	12,578,625–12,580,313	1,014	2	337	36.47	8.04
AcPRX27	Aco003045	LG06	13,446,497–13,448,722	999	4	332	36.19	4.99
AcPRX28	Aco021646	LG06	3,277,357–3,281,208	999	4	332	36.19	8.39
AcPRX29	Aco004906	LG07	711,110–714,125	987	4	328	34.98	6.8
AcPRX30	Aco014483	LG07	13,942,612–13,950,563	1971	14	656	73.27	5.57
AcPRX31	Aco014484	LG07	13,930,364–13,942,392	2,229	9	742	82.31	8.49
AcPRX32	Aco014486	LG07	13,917,358–13,919,006	987	4	328	36.41	7.06
AcPRX33	Aco016652	LG08	10,748,165–10,759,910	1989	8	662	70.55	4.87
AcPRX34	Aco022304	LG08	12,736,030–12,741,485	1,668	7	555	59.63	5.83
AcPRX35	Aco008544	LG09	630,761–632,783	990	4	329	35.57	5.06
AcPRX36	Aco008990	LG09	12,648,397–12,649,951	987	3	328	36.57	8.43
AcPRX37	Aco009079	LG09	13,183,005–13,184,814	1,026	3	341	37.16	4.9
AcPRX38	Aco015772	LG09	10,764,795–10,767,222	999	4	332	35.59	7.5
AcPRX39	Aco009743	LG10	64,021–70,344	1,521	7	506	55.56	9.17
AcPRX40	Aco010007	LG10	2,135,816–2,138,014	951	3	316	34.21	5.89
AcPRX41	Aco010009	LG10	2,138,049–2,158,166	2,760	11	919	98.4	7.93
AcPRX42	Aco020332	LG10	12,894,588–12,902,324	1,092	5	363	39.52	8.71
AcPRX43	Aco020334	LG10	12,882,382–12,885,182	1,044	4	347	38.36	5.17
AcPRX44	Aco005737	LG11	12,540,906–12,548,249	1,545	5	514	56.57	9.92
AcPRX45	Aco016519	LG11	383,223–387,691	1,011	4	336	36.65	8.08
AcPRX46	Aco012522	LG13	1,786,966–1,789,080	957	4	318	34.11	6.05
AcPRX47	Aco012524	LG13	1,805,368–1,809,238	960	4	319	34.05	8.09
AcPRX48	Aco013666	LG13	11,351,548–11,352,919	1,050	2	349	38.04	6.67
AcPRX49	Aco006432	LG14	2,360,239–2,362,650	999	4	332	35.12	4.61
AcPRX50	Aco014874	LG14	344,806–346,770	1,053	4	350	38.81	8.46
AcPRX51	Aco013384	LG15	10,676,947–10,698,878	2,886	12	961	103.55	5.97
AcPRX52	Aco006230	LG16	7,715,989–7,722,953	1,104	3	367	39.66	8.85
AcPRX53	Aco021127	LG16	508,904–513,657	969	4	322	34.98	8.33
AcPRX54	Aco026779	LG16	183,852–190,883	999	5	332	35.78	4.57
AcPRX55	Aco003198	LG17	1,062,168–1,065,621	1,083	3	360	38.62	8.47
AcPRX56	Aco003200	LG17	1,070,224–1,073,010	1,083	3	360	38.71	8.58
AcPRX57	Aco003320	LG17	1,983,887–1,985,624	1,002	4	333	36.02	5.1
AcPRX58	Aco001617	LG18	9,253,311–9,270,632	2,364	12	787	83.69	4.95
AcPRX59	Aco001618	LG18	9,248,026–9,252,038	1914	6	637	67.86	4.52
AcPRX60	Aco008430	LG19	10,318,327–10,320,385	1,020	4	339	38.22	8.53
AcPRX61	Aco008465	LG19	10,590,430–10,593,279	1,002	4	333	36.69	6.94
AcPRX62	Aco015271	LG20	10,156,870–10,159,414	1,068	2	355	37.94	8.92
AcPRX63	Aco019704	LG20	8,345,185–8,348,513	1,014	4	337	35.72	4.7
AcPRX64	Aco028441	LG20	8,837,469–8,839,195	303	2	100	11.33	9.96
AcPRX65	Aco009344	LG22	7,804,594–7,809,328	948	4	315	33.75	6.06
AcPRX66	Aco017479	LG22	1,177,709–1,189,266	3,174	9	1,057	118.18	5.84
AcPRX67	Aco007303	LG23	3,248,931–3,253,068	981	4	326	34.91	6.1
AcPRX68	Aco012993	LG25	1,550,545–1,551,925	975	4	324	35.04	6.07
AcPRX69	Aco012994	LG25	1,544,211–1,545,775	984	4	327	35.19	7.54
AcPRX70	Aco030748	scaffold_1315	7,476–13,988	1,059	4	352	37.48	4.73
AcPRX71	Aco028733	scaffold_1328	5,042–7,342	981	4	326	35.37	4.87
AcPRX72	Aco029135	scaffold_1464	25,017–29,639	1,227	3	408	43.69	6.23
AcPRX73	Aco029860	scaffold_1666	5,369–16,413	1758	8	585	63.71	8.93
AcPRX74	Aco027813	scaffold_1838	5,808–8,224	999	4	332	35.12	4.61
AcPRX75	Aco031666	scaffold_2216	6,898–8,468	966	3	321	34.55	7.07
AcPRX76	Aco029389	scaffold_2281	5,013–7,429	999	4	332	35.12	4.61
AcPRX77	Aco031596	scaffold_2950	101–3,231	693	3	230	25.96	9.4
AcPRX78	Aco025498	scaffold_691	13,170–16,131	1,056	3	351	38	10.28

### Classification and phylogenetic analysis of *AcPRXs*

**Figure 4:**
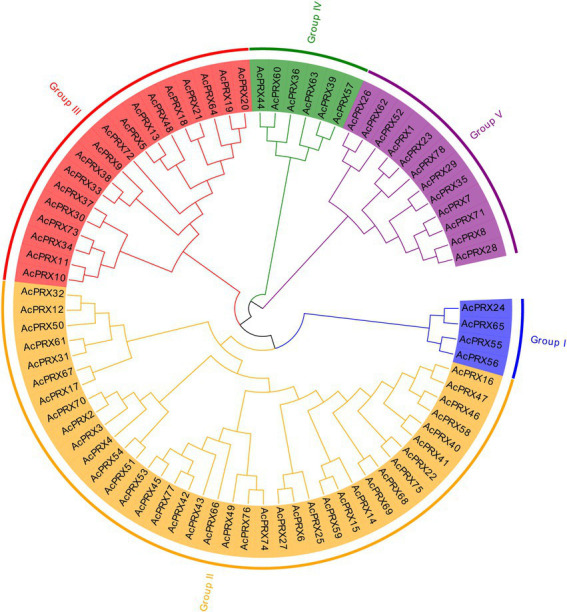
Classification and phylogenetic analysis of pineapple *PRX* genes. There were a total of 78 *PRXs*, all of which were used to construct the phylogenetic tree with the neighbour-joining (NJ) method and 1,000 bootstraps.

Multiple sequence alignment was conducted to confirm the similarity and homology of *AcPRX* genes, and MEGA 6.0 was used to generate a phylogenetic tree. The results of the phylogenetic analyses suggested that AcPRXs could be split into five subgroups based on genetic distance (Figure 4). Among these five subgroups, subgroup II was the largest (38 *AcPRXs*) and subgroup I was the smallest (4); subgroups III, IV, and V contained 18, 6, and 12 AcPRX genes, respectively.

### Conserved exon–intron and motif structure in *AcPRXs*

**Figure 5:**
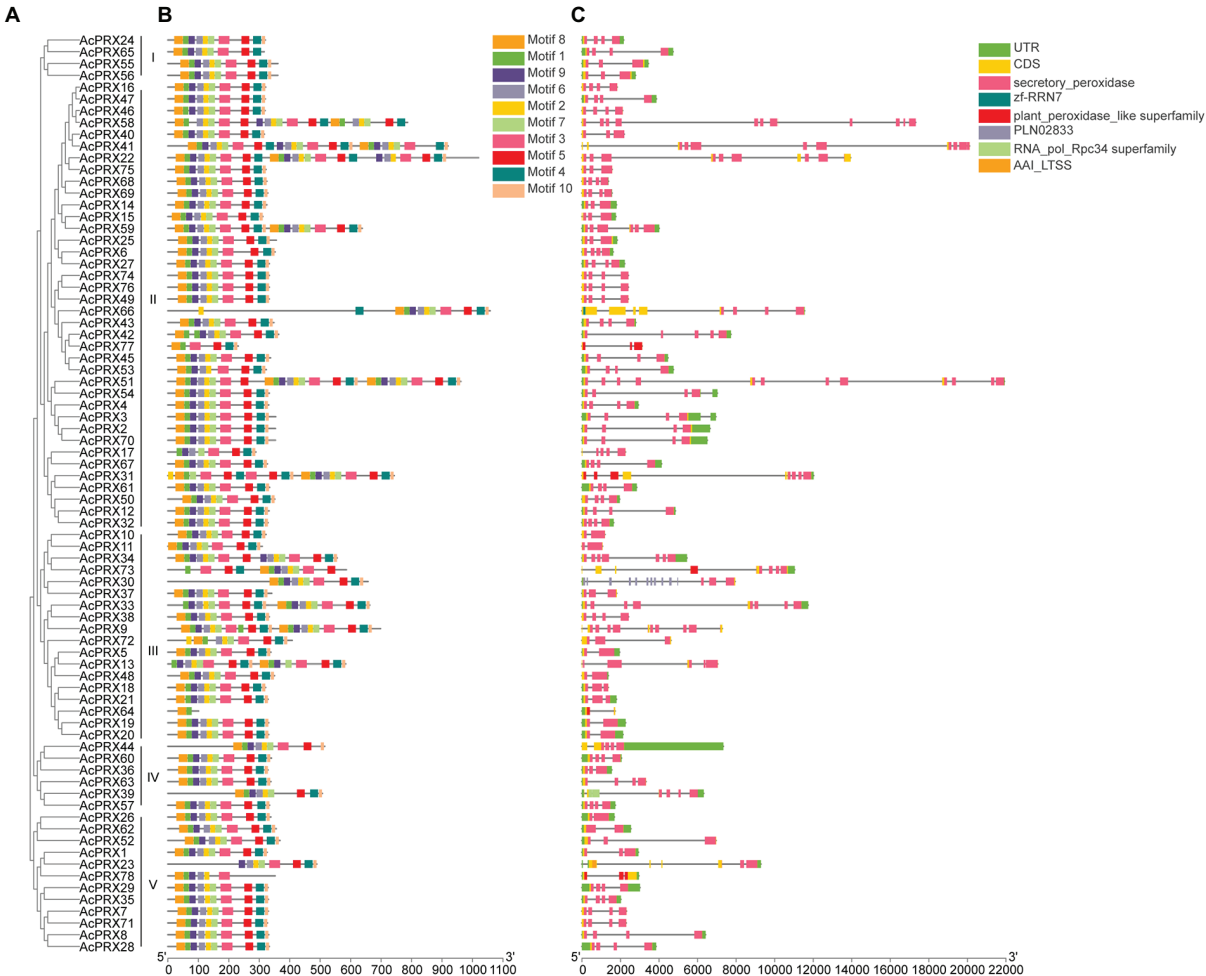
The conserved exon-intron structure and motif analyses of *AcPRXs*. **(A)** The full-length amino acid sequences of 78 AcPRX proteins were used to construct an unrooted neighbour-joining phylogenetic tree with 1,000 bootstrap replicates. **(B)** Organization of conserved motifs in AcPRXs. There were a total of 10 inferred motifs represented by different coloured boxes. Detailed information for each motif can be found in Table 2. **(C)** Exon-intron structure of *AcPRXs*. Grey lines indicate introns and yellow boxes indicate exons. The blue boxes represent 5′ and 3′ untranslated regions. The scales at the bottom illustrate the size of introns and exons.

Genomic DNA and whole-length cDNA sequences were aligned for the *AcPRXs*, demonstrating the exon–intron structure of each gene (Figure 5C). Of the *AcPRX* genes, 9 (11.54%) had one intron, 14 (17.95%) had two intron, 35 (44.87%) had three intron, 5 (6.41%) had four intron, 2 (2.56%) had five intron, 3 (3.85%) had six intron, 2 (2.56%) had seven intron, 2 (2.56%) had eight intron, 1 (1.28) had nine intron, 2 (2.56%) had 10 intron, 2 (2.56%) had 11 intron, and 1 (1.28%) had 13 intron. Genes with no intron were not observed. The highest proportion of *AcPRXs* had 2–5 exons (80.77%). In general, *AcPRXs* in the same subgroup generally had similar intron–exon structure, demonstrating the phylogenetic relationship between genes (Figures 5A,C).

The main structural characteristics of *AcPRXs* comprised 10 conserved motifs identified by the MEME database based on the phylogenetic relationship between genes (Figure 5B). The sizes of the conserved motifs ranged from 11 to 36 amino acids (Table 2). The results from InterPro database analyses indicated that motifs 1–2 were all in the conserved region of the hame peroxidase superfamily, which were characteristic domain of the peroxidase family. The different combinations of those motifs could result in functional differences. AcPRX proteins grouped together in the phylogenetic tree possessed similar motifs, and may therefore have similar functions to one another.

**Table 2:** List of the identified motifs in AcPRX proteins.

Motif	Best possible match	Width	InterProScan analyse
1	KDPRMAASLLRLHFHDCF	18	Peroxidase active site
2	VVSCADILALAARDSVV	17	Haem_peroxidase
3	SQLISKFASKGLSLTDLVALSGAHTIGRAHCSSFSN	36	Peroxidases heam-ligand binding site
4	YAANQSAFFADFAAAMVKMGNIGVLTG	27	Haem_peroxidase
5	TPNTFDNAYYKNLLAGKGLLTSDQAL	26	Haem_peroxidase
6	SLRGFDVIDDIKAAVEAACPG	21	None predicted
7	LAGGPSWTVPLGRRDGTTSSA	21	Haem_peroxidase
8	AAQLSPGFYDSTCPNAESIVRSVVEKAVA	29	Haem_peroxidase
9	VRGCDASVLLDSTPTNTSEKB	21	None predicted
10	GEIRKNCRVVN	11	None predicted

### Chromosomal locations and gene duplication analysis of *AcPRXs*

**Figure 6:**
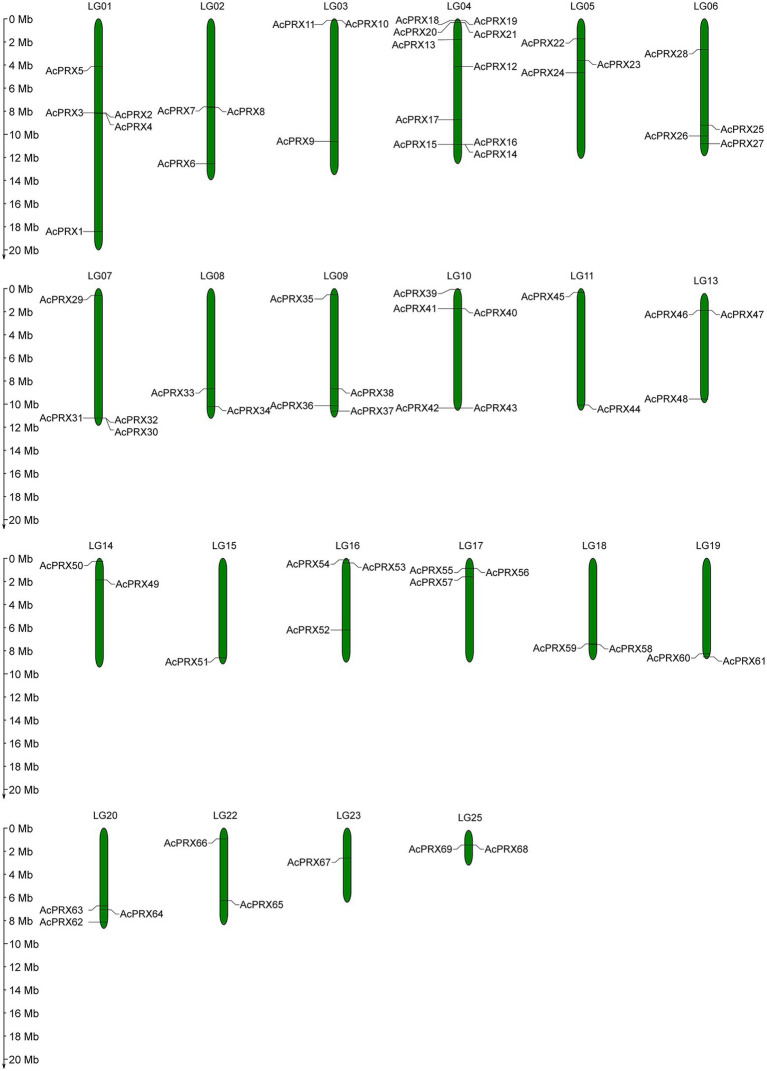
Chromosomal distribution of *AcPRXs*.

The identified *AcPRX*s were distributed across 22 chromosomes in the pineapple genome (Figure 6). The largest number of *AcPRXs* on a single chromosome was 10 (chromosome LG04), in comparison to the single *AcPRX* located on chromosome LG23 and LG15. Most were distributed on both sides of the chromosome. There were also nine *AcPRXs* that were on unannotated chromosomes. The most widely distributed was subgroup II, which contained 38 *AcPRXs* located across 17 chromosomes; members of subgroup I were distributed among the fewest chromosomes (three). In all, *AcPRXs* were widely distributed among chromosomes.

**Figure 7:**
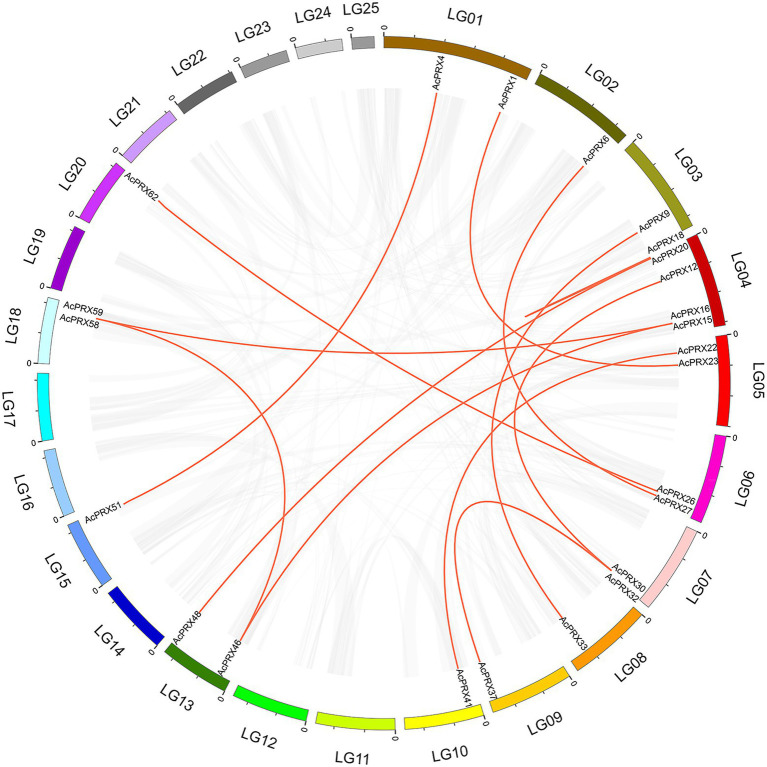
Analysis of gene duplication events leading to extant *AcPRXs*. All synteny blocks are represented by grey lines, and duplicated PRX gene pairs are connected by red lines.

Over the course of evolution, genome-wide duplication, segmental and tandem duplication have promoted the formation of gene families (Cannon et al., 2004; Li et al., 2020a). In order to investigate the effect of duplication on the *AcPRXs* gene family, we analysed duplication events among *AcPRXs*. Using the definition of Holub (Holub, 2001), two or more homologs within a chromosome area of 200 kb were considered tandem duplicates. A total of 22 *AcPRXs* were clustered into 17 tandem duplication event regions: *AcPRX2/3, AcPRX2/4*, *AcPRX3/4*, *AcPRX7/8*, *AcPRX10/11*, *AcPRX14/15*, *AcPRX14/16*, *AcPRX18/19*, *AcPRX18/20*, *AcPRX18/21*, *AcPRX19/20*, *AcPRX19/21*, *AcPRX20/21*, *AcPRX40/41*, *AcPRX55/56*, *AcPRX58/59*, and *AcPRX68/69* (Figure 6). Groups II and III had the highest number of tandem genes, containing 12 and 6, respectively. Group IV had no tandem genes. All tandem duplication events were distributed on eight chromosomes (LG 01, LG 02, LG 03, LG 04, LG 10, LG 17, LG 18, and LG 25). LG 04, and LG 01 had eight and three clusters of tandem duplicates respectively, and the other six chromosomes containing *AcPRX* tandem duplicates had just one cluster each, which suggested that a high tandem duplication frequency exists in the *AcPRX* gene family. In addition to the tandem duplication events, there were 15 segmental duplication events identified involving 24 *AcPRX* genes: *AcPRX59*/*16*, *AcPRX58*/*46*, *AcPRX46*/*15*, *AcPRX59*/*15*, *AcPRX30*/*12*, *AcPRX32*/*37*, *AcPRX30*/*37*, *AcPRX22*/*41*, *AcPRX20*/*18*, *AcPRX33*/*9*, *AcPRX48*/*20*, *AcPRX51*/*4*, *AcPRX27*/*6*, *AcPRX62*/*26*, and *AcPRX23*/*1* (Figure 7). The segmental duplicates were spread across 13 chromosomes and all pineapple AcPRX subgroups except for subgroup I and IV. According to the karyotype evolution analyse results of pineapple in the monocots (Ming et al., 2015), there were seven genome-wide duplication events involving in 19 AcPRX genes among all the segmental duplication events (Supplementary Table 1), which suggested that the genome-wide duplication events occupy an important role in promoting pineapple segmental duplication events. These results suggest that tandem and segmental duplication events may have played approximately equal roles in promoting the expansion of the *AcPRX* gene family.

To better understand the evolution course for AcPRX gene family, the divergence time of the AcPRX gene family was estimated based on *K*s values (Supplementary Table 1). The divergence time for the 17 tandem duplication events ranged from 3.46 to 274.13 mya, and that for the 15 segmental duplication events ranged from 58.07 to 185.44 mya, which suggested that the AcPRX gene family is generated through a long evolutionary journey.

### Differential expression *AcPRXs* between tissues and developmental stages

**Figure 8:**
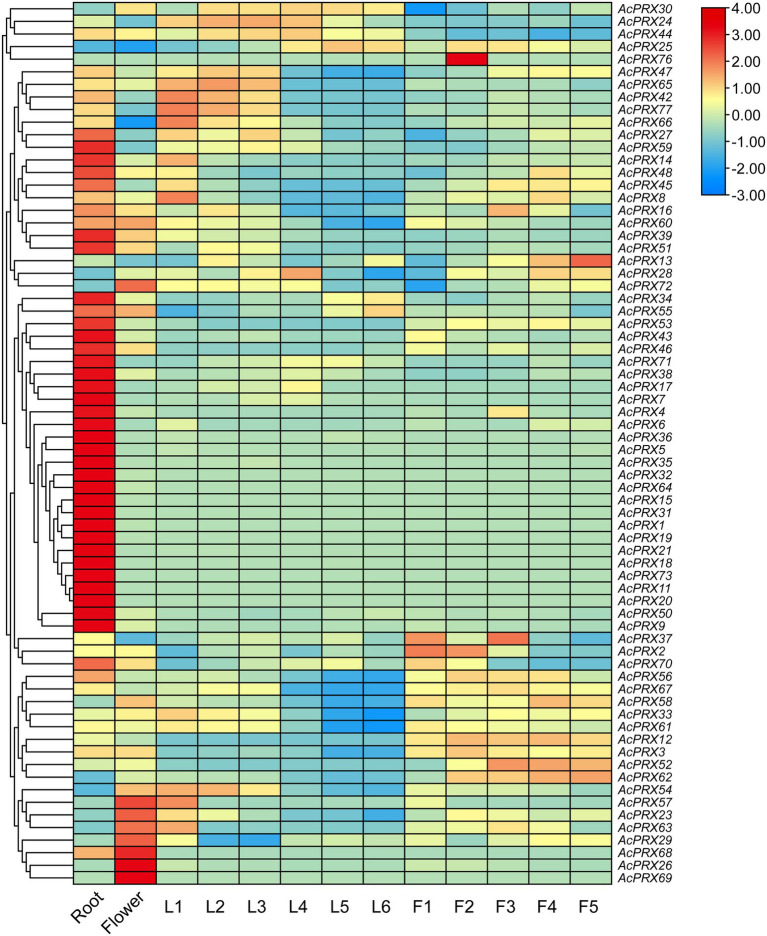
Expression pattern analysis of *AcPRXs* in different tissues and developmental stages in pineapple. Gene expression is shown as the log_2_ transformation of fragments per kilobase of exon per million mapped fragments (FPKM) values. *AcPRXs* are clustered based on their phylogenetic relationships. Relative expression values are represented by colour, with red showing high expression and blue showing low expression.

Expression profiles of *AcPRX* genes were obtained from PGD and analysed to identify differences between tissues including the root, flowers, and expanded leaves (from leaf base to leaf tip, segments referred to as L1–L6) and in fruits from different developmental stages (F1–F5; Figure 8; Supplementary Table 2). There were transcripts of 65 *AcPRX* genes in the root, 90.77% of which were expressed at high levels (|log_2_| ≥ 1). In the flower, there were 60 *AcPRX* genes expressed, with 85% highly expressed. In addition, *AcAPX15*, *−19*, *−31*, *−32*, *−64*, *−68*, and *−69* were only expressed in the root and flower, not in the leaf or fruit. There were 56, 54, 54, 47, 45, and 53 genes expressed in L1–L6, respectively, with 82.14, 77.78, 81.48, 85.11, 86.67, and 83.02% highly expressed (|log_2_| ≥ 1). Expression levels of *AcPRX8*, *−23*, *−33*, *−42*, *−54*, *−60*, *−61*, and *−66* decreased from the leaf base to the tip. Twenty genes were not expressed in any part of the leaf (Supplementary Table 2). At developmental stages F1–F5 in the fruit, 65.96% (31/47), 80.39% (41/51), 80.39% (41/51), 80% (40/50), and 82.69% (43/52) respectively, of the genes were highly expressed (|log_2_| ≥ 1). The expression of *AcPRX2*, *−9, −26*, *−60*, and −*61* decreased steadily from F1 to F5. Twenty-three genes were not expressed in the fruit at any stage (Supplementary Table 2). It is worth noting that, except for in the root, there were more downregulated than upregulated *AcPRX* genes in every tissue. Furthermore, the 27 genes that were upregulated in the root were downregulated in the other tissues. These results show that most of the genes were differentially expressed between tissues, potentially explaining the functional diversity in different tissues. Although there were a variety of expression patterns, closely related genes showed similar expression patterns in different tissues. Most of the genes were downregulated, suggesting that *AcPRXs* may have a predominantly negative effect on pineapple tissues and development. These tissue expression profiles of *AcPRX* genes will provide clues for further understanding pineapple growth and fruit development.

### Expression profiles of *AcPRXs* during postharvest storage

**Figure 9:**
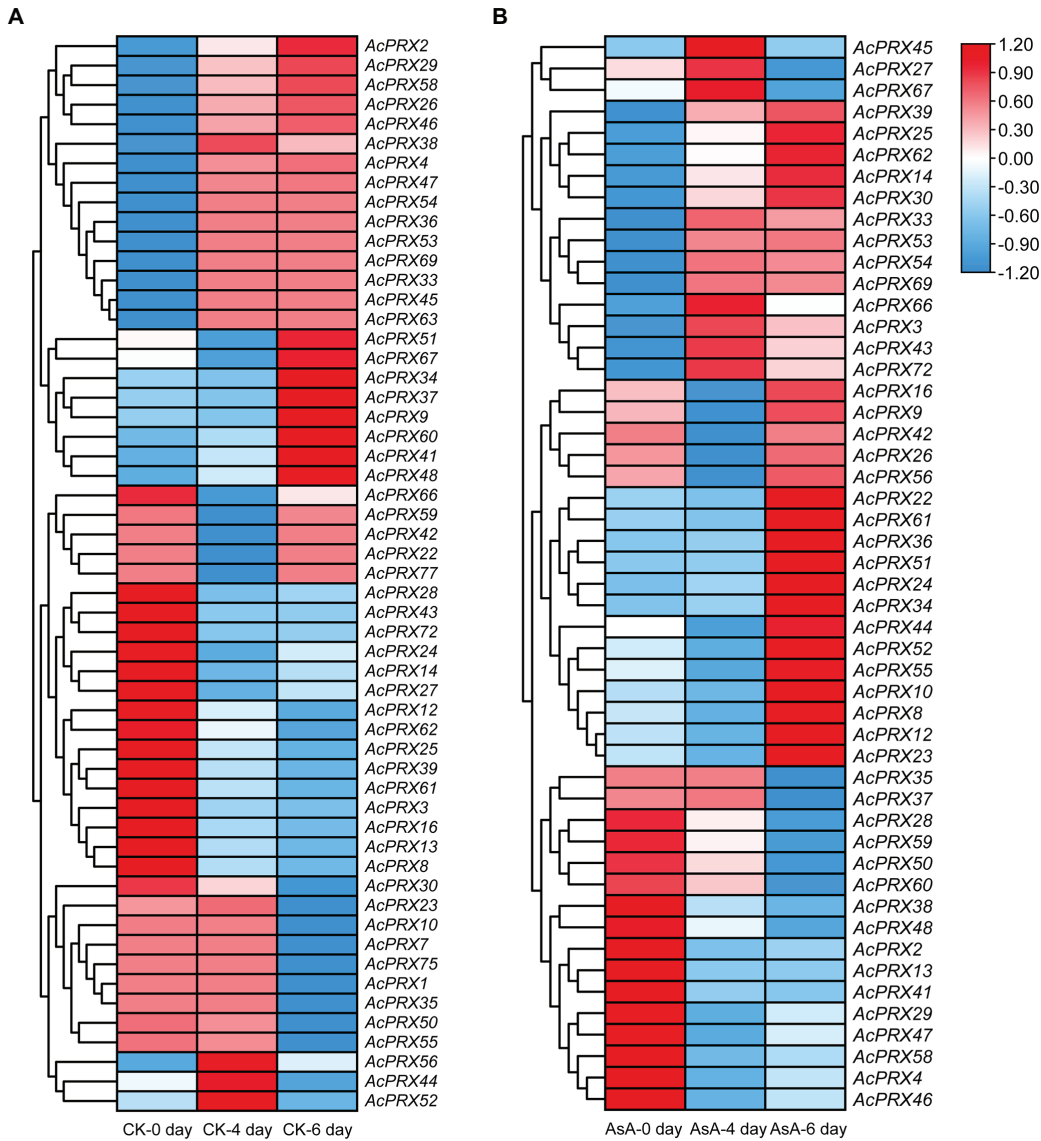
Expression patterns of *AcPRXs* in the control group **(A)** and after AsA treatment **(B)**. The transcript data were generated from three replicates. Log_2_ fold change values were used to create the heatmaps. *AcPRXs* are clustered based on their phylogenetic relationships. Relative expression values are represented by colour, with red showing high expression and blue showing low expression.

Peroxidases scavenge excessive ROS, and ROS is associated with the occurrence of IB. We therefore analysed the expression of *AcPRXs* at different timepoints after storage and in the control group compared to the group treated with AsA using RNA-Seq data. Each storage timepoint was compared to the 0 day timepoint. In the control group, 29.49% of the *AcPRX* genes were not expressed at any storage stage: *AcPRX5*, *−6*, *−11*, *−15*, *−17*, *−18*, *−19*, *−20*, *−21*, *−31*, *−32*, *−40*, *−49*, *−57*, *−64*, *−65*, *−68*, *−70*, *−71*, *−73*, *−74*, *−76*, and *−78*. However, the expression of the other 55 genes varied significantly between timepoints (Figure 9A; Supplementary Table 3). Genes that were up- or downregulated accounted for 41.82 and 56.36%, respectively, of the expression genes. *AcPRX44*, *−52*, and *−56* were significantly increased at 4 days of storage, but decreased at 6 days; *AcPRX22*, *−42*, *−59*, *−66*, and *−77* showed the opposite trend at the same timepoints. These results demonstrate the participation of *AcPRXs* in the pathway involved in IB response in the postharvest storage stage. Many more *AcPRXs* were downregulated than upregulated, further suggesting that they may have primarily negative effects on IB.

Ascorbic acid-treated samples were compared with the control group at each corresponding timepoint (Figure 9B; Supplementary Table 3). Genes that were not expressed in the CK group were typically not expressed in the AsA treatment group, except for *AcPRX57*, *−68*, and *−71*. *AcPRX27*, *−45* and *−67* were significantly upregulated at 4 days, but expression levels decreased at 6 days. In addition, more genes were upregulated (49.06%) than were downregulated (30.19%) after AsA treatment, whereas the opposite trend was observed in the CK group. Based on these results, we speculate that *AcPRX* genes participate in the response to AsA that delays the spread and deterioration of IB.

### Validation of PRX gene expression *via* qRT-PCR

**Figure 10:**
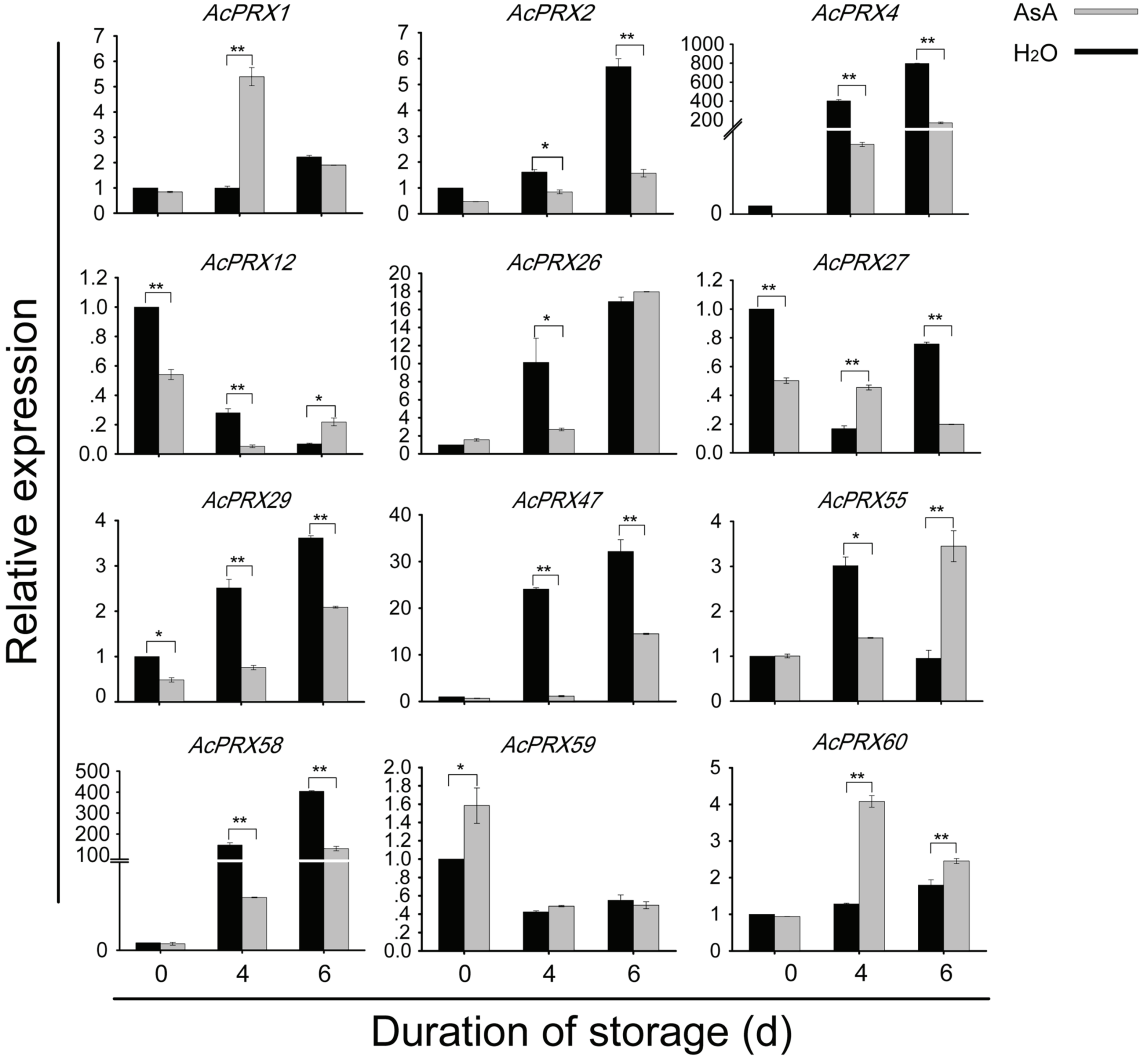
Expression of AcPRX genes during pineapple postharvest storage after treatment with ascorbic acid (AsA) or a water control. ^*^p ≤ 0.05, ^**^p ≤ 0.01 (Student’s *t*-test).

Quantitative real-time PCR was conducted to verify the consistency of the RNA-Seq data and to confirm the importance of *AcPRX* genes in regulating the resistance of pineapple to IB. Twelve genes (*AcPRX1*, *−2*, *−4*, *−12*, *−26*, *−27*, *−29*, *−47*, *−55*, *−58*, *−59*, and −*60*), which showed different expression patterns in the RNA-Seq data, were selected for validation with qRT-PCR (Figure 10; Primers for qRT-PCR in Supplementary Table 3). The results showed that *AcPRXs* responded to the occurrence of IB, and expression levels of the 12 *AcPRX* genes were consistent with the RNA-Seq data. Expression levels of these genes were significantly altered by exposure to AsA. We found that eight genes were continuously upregulated in the CK group. However, AsA significantly delayed higher expression of *AcPRX2*, *AcPRX4*, *AcPRX26*, *AcPRX29*, *AcPRX47*, and *AcPRX58* and enhanced expression of *AcPRX1* and *AcPXR60*, especially at 4 days. In total, the results supported that *AcPRXs* negatively regulate IB, and that AsA can alter *AcPRXs* expression.

## Discussion

Due to its unique taste and economic and industrial value, pineapple holds a vital position in tropical and subtropical countries (Zhang et al., 2015). IB, a physiological disorder in pineapple fruits, has long been a major challenge that hinders the development of the pineapple industry. IB occurs when excessive ROS damage the membrane, causing phenolic compounds to be oxidated into quinones by PPO (Richard, 2000). ROS, including free radicals such as superoxide (O^2−^) and hydroxyl (-OH), and non-radicals like singlet oxygen (1O_2_) and hydrogen peroxide (H_2_O_2_; Choudhury et al., 2017), are necessary for IB to occur. In the present study, AsA, a ROS scavenger and oxidant, was firstly used to treat pineapple fruits prior to storage. AsA significantly decreased the IB index and the incidence of IB (Figure 2B) and reduced the disease grade level (Figure 2A). The results illustrated that AsA can significantly delay the spread and progression of IB in pineapple fruits compared with water-treated control fruits. H_2_O_2_ and MDA are commonly used as indicators of ROS content and the severity of cell membrane injury (Zhang et al., 2015). We here found that AsA-treated pineapples had much lower levels of MDA and H_2_O_2_ than fruits in the control group. PPO activity, an indicator of IB occurrence (Ko et al., 2013), was decreased at all timepoints after AsA treatment (Figures 3A,C,D). The results suggested that AsA could scavenge excessive ROS and reduce MDA content, protecting membranes from damage and further delaying progression of IB. Due to the key role of compartmentalization between PPO in the plastid and polyphenols in the vacuole in protecting pineapple against IB occurrence, scavenging excessive ROS is an effective means to prevent IB.

Interestingly, we found the storage period from 3 to 6 days was found to be the transitional period from the translucency symptoms to browning of the fruit tissue (Figure 2A). During this time period, all damage indicators reached a plateau after AsA treatment, and activities of PRXs were much higher in the AsA-treated group than in the control group at the same timepoint (Figure 3). The results suggest that this period may be a key to studying the mechanism underlying IB. Members of the peroxidase family play very important roles in excessive ROS scavenging in plants, and we hypothesize that PRXs may be involved in this pathway. This shared function suggests that members of the PRX family may regulate the prevalence of IB in pineapple *via* an unknown mechanism.

Class III PRXs are a key in maintaining ROS homeostasis in plants (Kim et al., 2011, 2013; Maria Kidwai et al., 2019; Zhang et al., 2019; Su et al., 2020). We here identified 78 PRXs in pineapple, meaning the PRX family is smaller in pineapple than in cassava (Wu et al., 2019), Chinese Pear (Cao et al., 2016), maize (Wang et al., 2015), and wheat (Yan et al., 2019). In pineapple, 76.92% of PRXs have a molecular mass ranging from 30 to 50 kDa, which is similar to the PRX families in other plants (Barcelo et al., 2007; Almagro et al., 2009). All of the AcPRX genes have more than one exon (Table 1), consistent with those in maize (Wang et al., 2015) and Chinese pear (Cao et al., 2016). The results of gene structure and motif composition analysis in the AcPRX family thus demonstrate the general conservation of PRX families throughout the plant kingdom.

Gene families are mainly formed though three methods: whole-genome duplication, tandem duplication of individual genes, and/or segmental duplication of multiple genes (Freeling, 2009). To better understand the duplication events giving rise to the AcPRX family, we analysed the chromosome locations of each family member. The results showed that AcPRX family members are distributed across all pineapple chromosomes (except for chromosomes 12, 21, and 24), consistent with the chromosomal locations of PRXs in rice (Passardi et al., 2004), maize (Wang et al., 2015), and Arabidopsis (Tognolli et al., 2002). Subsequently, we identified a total of 22 *AcPRX* genes derived from 17 tandem duplication events (Figure 6) and 24 *AcPRX* genes derived from 15 segmental duplication events (Figure 7). Using RNA-Seq data, we found that most of the duplicated *AcPRX* genes were expressed during all developmental stages. We also identified distinct *AcPRX* expression patterns in different tissues, fruit developmental stages, and during the occurrence of IB by studying stored pineapple fruits over several timepoints after treatment with AsA or a water control. Among the 17 tandem duplicated *AcPRX* genes, 10 were upregulated in pineapple fruit during all five developmental stages, and 17 were upregulated in the root (Figure 8). During postharvest storage, five *AcPRX* genes were upregulated, but the expression patterns changed in response to AsA treatment (Figure 9). It has previously been shown that nearly all known plant PRX gene families have been expanded *via* gene duplication, and this diversity allows plants to respond to a range of biological processes and perform numerous biological functions even when there are limited gene resources (Cao et al., 2016; Yan et al., 2019). These results may partly explain why AcPRXs were expressed during all growth stages.

Using the expression profiles of *AcPRX* family members obtained from online RNA-Seq databases, we found that most *AcPRX* genes were downregulated across different tissues. These trends were similar to those of *PRX* genes in maize (Wang et al., 2015) and Chinese Pear (Cao et al., 2016), and implied that *AcPRX* genes may play a predominantly negative role in pineapple development (Figure 8). *AcPRX* gene expression was then correlated with different stages of IB incidence, and although various expression trends were observed, more *AcPRX* genes were downregulated than upregulated (Figure 9). This again suggested that members of the *AcPRX* gene family may widely participate in and negatively regulate the occurrence of IB. The number of upregulated *AcPRXs* increased in AsA-treated plants, which also showed a delay in the spread of IB. We further selected 12 *AcPRX* genes that demonstrated different expression patterns in the IB RNA-Seq data for validation with qRT-PCR. The expression patterns identified *via* qRT-PCR were consistent with the RNA-Seq data for all 12 *AcPRX* genes, confirming the high quality of the RNA-Seq data for the postharvest storage phase. In addition, expression patterns of the 12 *AcPRX* genes were altered in response to AsA treatment compared with the CK group; nearly all genes showed decreased expression, except for *AcPRX1*, *−59*, and *−60*. This suggests that most of the selected *AcPRX* genes negatively regulate pineapple resistance to IB. These results demonstrate that *AcPRXs* respond to IB occurrence and also play a vital negative regulatory function. Future studies are needed to elucidate how *AcPRXs* and AsA interact to influence ROS homeostasis and PPO activity, and ultimately how they regulate the resistance of pineapple to IB. This will be a key novel direction in furthering our understanding of the molecular mechanisms underlying IB resistance in pineapple.

In summary, this study clarified that exogenous AsA can function as a buffer solution to scavenge excessive ROS, effectively preventing the spread and deterioration of IB in pineapple. Through an observed plateau in ROS markers, we found that the third and sixth days in pineapple postharvest storage may be the vital timepoints for delaying IB. Furthermore, 78 AcPRXs were identified in pineapple and their basic physicochemical properties, phylogenetic classification, protein motifs, gene structures, chromosomal distribution, duplication patterns, and expression pattern characteristics were described. Based on transcriptome data and qRT-PCR results, we conclude that the *AcPRX* genes family plays an important role in negatively regulating IB occurrence. Our findings provide a new direction for studying the mechanisms of IB occurrence and contribute to develop IB-resistant varieties in pineapple.

## Data availability statement

The original contributions presented in the study are included in the article/Supplementary material; further inquiries can be directed to the corresponding authors.

## Author contributions

XH conceived and designed experiments, and edited the manuscript. KS and HG performed the partial experiments. ZL conducted the bioinformatic work and edited the manuscript. KH and WH contributed with valuable discussions. QY coordinated the project. All authors contributed to the article and approved the submitted version.

## Funding

This research was financially supported by National Key R&D Program of China (2018YFD1000500) and Natural Science Foundation of Hainan Province (320MS089).

## Conflict of interest

The authors declare that the research was conducted in the absence of any commercial or financial relationships that could be construed as a potential conflict of interest.

## Publisher’s note

All claims expressed in this article are solely those of the authors and do not necessarily represent those of their affiliated organizations, or those of the publisher, the editors and the reviewers. Any product that may be evaluated in this article, or claim that may be made by its manufacturer, is not guaranteed or endorsed by the publisher.

## Supplementary material

The Supplementary Material for this article can be found online at: https://www.frontiersin.org/articles/10.3389/fpls. 2022.953623/full#supplementary-material

Supplementary File 1 The full-length amino acid sequences of verified AcPRXs.

Supplementary Table 1 Duplication events and divergence time analysis results.

Supplementary Table 2 FPKM values of different tissue and development of *AcPRXs*.

Supplementary Table 3 FPKM values of different storage timepoint in AsA treatment and control group of *AcPRXs*.

Supplementary Table 4 Primers used in this study.

## Footnotes

^1^ https://rapdb.dna.affrc.go.jp/

^2^ https://www.arabidopsis.org/

^3^ http://smart.embl-heidelberg.de/

^4^ https://www.ncbi.nlm.nih.gov/Structure/bwrpsb/bwrpsb.cgi

^5^ http://web.expasy.org/protparam/

^6^ http://meme-suite.org/tools/meme

^7^ https://www.ebi.ac.uk/interpro/

^8^ http://gsds.cbi.pku.edu.cn/

^9^ https://sigmaplot.en.softonic.com
