# Supplementary material for: The class III peroxidase gene family is involved in ascorbic acid induced delay of internal browning in pineapple
Source: Front Plant Sci. 2022 Aug 3;13:953623. doi: 10.3389/fpls.2022.953623 (PMC9382127; doi:10.3389/fpls.2022.953623)
Supplement: Supplementary File 1 — The full-length amino acid sequences of verified AcPRXs. [file Data_Sheet_1.zip › Supplementary Material/Supplementary File 1.doc]

>AcPRX1

MEQHYLRGLVVGLVLLLALRIAEAGLSPNYYETSCPNVESIVRQAVEKKKNATVVTVPATLRLFFHDCFVEGCDASVLIASPNGDAEKDAPDNISLAGDGFDTVIKAKEAVEAECPGVVSCADILAIAARDVVALAGGPNFTVELGRLDGLISNASLVAGNLPGPDYNLANLTSLFGKHNLTVLDVVALSGAHTVGFAHCTRFADRLYNFSSTLPVDPSLNPVYAEQLEQVCPPHVDPTMAVNMDPVTPVTFDSYYYKNLVDGLGLFRSDEVLYTDNRTKSAVDDFAGNQSSFFAAFAHAMVKLGRVGVKAGKLGQIRRDCTTFN*

>AcPRX2

MSHPCSLLLALNLITITLALLSQRSQAQLSPSFYNSTCSNVSAIVRSTVQQAQTSDVRIYASLTRLFFHDCFVQGCDGSVLLVNSSTIQTEQDAVPNKNSIRGFNVVADIKTAVENACPGVVSCADILALTAEASVNLAGGPSWTVLLGRRDSTTANITAANNLPSPFDNLTTLEQKFAAVGLNDTDLVTLSGAHTFGRGQCRFVTPRLYNFSSTVNTDPTLNSTYATTLEQNCPQGGNASVLNNLDPITPNTFDSNYYINLQNHQGLFTSDQELLSDPNATTTASIVNNFAGNQSAFFDSFVQSMINMGNISPLTGSSGEIRTNCSVVNSGSGLELHIRIQHDSDKGSAAE*

>AcPRX3

MSHPRYLLLPLNLITITLALLSQRSQAQLSPSFYDCTCSDVSAIVRSTVQQAQTSDPRIYASLTRLFFHDCFVQGCDGSILLVDSSTIQSEQDARPNKNSARGFNVVDDIKTAVENACPGVVSCADILALAAEASVNLAGGPSWTVLLGRRDSTTANFTAAGNLPGPFDNLTTLQKKFSDVGLHDIDLVTLSGAHTFGRGQCQFFSNSRLYNFNGTGKPDPTLNSTYRTILEQNCPQGGNGSVLNNLDPITPNKFDGNYYINLQNHRGLFTSDQELLSDPNATTTASIVNNFAGNQRAFFNSFVQSMIKMGNISPLTGSSGEIRTNCSVVNSGSGLELHIQIRHDSDTGSAAE*

>AcPRX4

MAHTSLLLALSLITIPLALLFQGSQAQLSSTFYDTSCSNVSAIVRSVVQQAQTSDPRIYASLTRLFFHDCFVDGCDGSLLLDDSATIQSEKDATPNKNSARGFDVVDNIKTAVENACPGIVSCADILAITAEASVNLAGGPSWTVLLGRRDSTTANQAGANNLPSPFDSLSTLESKFSAVGLDDTDLATLSGAHTFGRAQCRFFSNRLYNFSGTGSPDPSLNPSYLTTLQQNCPQGGDGTVLNNLDPTTPNTFDNNYYTNLQNNRGLLQSDQELLSTSGASTASTVNSYAGSQSTFFQNFAQSMINMGNITPLTGSSGEIRSNCRKVNGS*

>AcPRX5

MGSRSYLLIFSSSLFFSIAILTPASPTTSALDVGFYRYSCPAVEAIVRQTVSQAVAQDPGLAAGLIRMHFHDCFVRGCDASVLLDSTPGNRAEKDSVANNPSLRGFPVIDAAKAAVEAACSRTVSCADILAFAARDAAFLAGGIDYPVPAGRRDGQVSRESEVLPNIPHPSFGARQLTANFAKKGLALDDMVTLSGAHSIGRSHCSSFDSRLYNFTTSRPQDPSMDPGFAAYLKTKCPTLTTPGSSDATDPTVPLDAVSPTTLDNQYYKNVVAHRGVLTSDQTLVASPATAGLVRFYAVERAAWAAKFAAAMAKMGSIGVLTGEEGEIRENCRVVNC*

>AcPRX6

MEKNMNVISCAEAVFLFLGCFLAIVSNLEAQDPSKLSLDYYSKSCPTAQQVVRSEMECAVRADPHNAAIILRLHFHDCFVQGCDGSVLLDDTVSMIGEKQADQNVNSLKGFDLVDKIKEKLEAECPGVVSCADLLAVAAKDAVVLVGGPYWDVPVGRLDSKKASLDQANTDIPTSDQGLATLISKFLAKGLSVTDMVALVGTTSYALIFHAARERSHTIGVSRCVNIRDRIYGDFQLTSKSEAASSLYLTKLKETCPARGGDDNTSPIDHVTPTIFDNAFFETLLHGEGLLNSDQEMYSSLIGFQTADIVAKYWADTAAFFKQFADSMVKMGNITNPEGGEVRKNCRFVNA*

>AcPRX7

LAAMIRMEKRLQLFLLSFTLSLLVSSPPGAAAVQLDPNYYANICPNLENLVRGAVVKMMSQTPVAAPATLRLFFHDCFVMGCDASIMIMSSDGSDEWHNPADFSLKAEGFETVLAAKAAVDGDLQCTNKVSCADILALATRDVVSLSGGPTWPVELGRYDGKVSLGSDVVLPSPDFNLDQLNGMFGSFGLTQTDMIALSGGHTIGASSCMFFYNRLYPGDPTVSPGFLLQLQQSCPNPLLDPNAFAFLDAATPLQFDNAYFRNLQQGMGLLNSDQVLFNDPRSQGTVNLFASNQSAFFSAFVDAMTRLGRLGTKTAVDGEIRRDCRFVN*

>AcPRX8

MESSNIFLLLSSLLSLLIFPSPPTAAAQLSQNYYSSICPNVESLVHTAVAQKLQLSFAAAPGTLRLFFHDCFVRGCDGSVLLAAANGDDENHSGADLTLSPEAVDTVNRAKAAVDGDPQCTNKVSCADILAIAARDVVSLTGGPYYQVELGRLDGRVTSKANVKHNLPGPGYNLDQLNAVFAANGLTQTDMIALSGAHTIGVTHCDKFIRRIYRFKQPLAFNPPMNLTFLRQMRQICPLNYNPSTFAMLDPATPFKFDNAYYQNLQKEMGLLASDQILFTDQRSRGLVNWFASNQTAFEEAFVAAMTKLGRVGVKNATEGEIRRVCTAVN*

>AcPRX9

MVVLWVLERGEIERERGERREMDRGQMLAATVLCFLLSNIISSEAELQVGFYNYICPAAESIVHDEVKRAVSYNPGFAAGLLRMHFHDCFVRGCDGSVLIDSTPFNEAEKDGPPNKSSLRGFEVVDNIKSQLEAVCQGKVSCADILAFAARDSVALTGGPSYDVPAGRRDGRISQAADTKDLPAPTFGLANLTAFFAKKNLTQEDMITLSGRSHCSFFDFRLYNFSSNMVQDPSLDPSYAELLKHECPKDSNDPNLVVPLNTLPPNAFDSSYYRAVLEGRGLFVSDQALLSTPESAALVQQYAADACFFKKKFAEAMGKMGQIEVLTGSSGEIRANCRELQDGYTSKMDTHQRAKNMALARGEILEAQLQFGFYDSICPEAESIVKDEVSKAVGANPSFAAALLRMHFHDCFVRGCDASVLIDSTSSNTAEKDAPPNTSLRGFDIVDGAKSRLEEVCGGIVSCADILAFAARDSIVLSGGLSYQVPAGRRDGRVSTAQETNGNLPPPTFNIDQLIQIFTLKGFTQEEMVTLSGAHTIGVSHCSSFSSRLYNFSSTMSQDPDLDPTYGAELMQLCPQGSTTNSGLIVPMDPYSPNSFDTAYYNNVLANQGLFTSDQALLSTDITAGQVIEYANNTDIFYADFAAAMVKMGQIEVLTGSLGEIRKHHQKLHTFEVQPLEPYLEELLGVGLGELGLDLVH*

>AcPRX10

ISIVELVIIYLCRLLNMVRVLLFVLLLSVPLASADLAVGFYDSSCPQAESVVRAVVAKHFHADHSIAAAFLRLYFHDCFVRGCDASILIDGTNTTDSEKSAFPNKTVRGYEIIDDIKQNLESQCPSTVSCADVIALATRDVVSLARGLHYAVPTGQRDGLVSNPANVSLPGPSSPMSVIQQAFGPKGFTLEETVVLLGAHTVGSVHCAFIQPDPSMDARLKRRLAKLCRGGDNTTTAFLDQATPFVVDNRFYKQVMRNKGVMGIDQELGSDNSTAPIVRRLAKDREGFLNKFTDVLVKMGSLDVLVGSEGEIRTNCRAFNN*

>AcPRX11

LADFRVGFYNATCPNAESIVQSVVAEHFAADPSVPAALLRMYFHDCFVRGCDASILISPTKKKTTERSAGPNQTVRGFDIIDEAESSLETQCPGNVSCADIIALAVRDAVALAGGPKYDVPTGRLDGLISNPNDVNLPGPQLTVSQALAFFTNKGFTLDDMVVLLGGHTVGVAHCTFFRDRLGNFQGTGQPDPTMDPALLAKLKETCGPRAKPLKSDPTAFLDQNASFAIDNSYYSQIMQKRGVMQIDQELADDNSTAATVQGLAADAAGFARRFADALVRLGSVEIKQGSDGEIRRSCQAFNTRSTKP*

>AcPRX12

MASKTLLVLLLPFNLVLFLSILAEAQLLQVGFYSKTCPNVEDIVRKEMTEILSVAPSLAGPLLRLHFHDCFVRGCDGSVLLDSTKNNVAEKEADPNKSLRGFGVIERVKAKVEKACPNTVSCADILALVARDAVWLSKGPSWPVWTGRRDGRVSLATETKQLPSPNANITRLISNFAAKGLSVKDLAVLSGGHTLGTSHCSSFSERLYNFTGKDNLHDVDPALDKEYVPKLRARCSLTDNTTIVEMDPGSFRTFDTSYYKYVAKRRGLFHSDASLLTNSETKAYVQRHADGLTAEFFQDFGDSMINMGNVAVLTGNQGEIRKKCYVVNKIN*

>AcPRX13

MSVSTDTRAVAHDPGIAAGLIRMHFHDCFVRGCDASVLLDSTPGNCAEKDSVANHPSLRGFPVIDTAKAAVEAACRRTVSCADILAFAARDAAFLAGGIDYPIPAGRRDGPSFSACQLTANFAKKGLALDDMIALSGAHSIGRSHCSSFGSRLYNFTTNRLQDPSMDPRFAAYLKTKCPTLTTPGSSDAIDPTVPLDAVSPTTLDNQYYKNVVAHRGVFTSDQTLAASPETAGLVRFYAAERAAWAAKFAAAMVKVGSIEVLTGEEGEIRENCRVSLVMGLLIFSSSLLFSIGILTLASPTTSTLDVGFYRYSCPAAEAIVRQTVSQAVAQDPGLAAGLIRMHFHDCFVRGCDASVLLDSTPGNRAEKDSVANNPSLRGFPWRPRAAGGIDYPVPAGRRDGRVSRKSEVLPNLPHPSFGARQLTANFAKKGLAVEDMVTLSGAHSIGRSHCSSFDSRLYNFTAGQPQDPSMDPRFAANLKTRCPTLTTPGSSDATDPTVPLDAVSPTTLDNQYYKNVVAHRGVLTSDQTLVASPVTAGLVRFYAAEREAWAAKFAAAMAKMGSIEVLTGEEGEIRENCRVVNC*

>AcPRX14

MATTQTLFTFFVAAAVVAFAAPTSGQLTLDHYDHVCPQALPTIKLLVDAAVGIEPRIGASLVRLHFHDCFVNGCDASVLLDDTPTMKGEKMAKPNNNSLRGFDMIDQIKTAVNIVCFGNVVSCADILAVAARDSIVALGGMPYDVLLGRQDAMTASVDAANAAIPNPLLDLPALISKFQSNNLTLQDLVVLSGAHTLGVARCTTFRDRIYNETSTIDPDFAAALQAQCPRTGDDDVPHSLDETPAKVDTEYFTGLTQRKGLLHTDQQLFQGDGSDADSLVKLYSENSDKFWADFGAAMVKMGNLSPLTGTDGEVRENCRVVNGD*

>AcPRX15

MATATFGTFTSVRGQLDPCFYDEVCPKALPAIKMIVEQAIEQEPRMGASLLRLHFHDCFVNGCDGSVLLDDTPTFTGEKNAAPNKNSIRGFDVVDKIKEAVNSACNGNVVSCADILAVAARDSVVALGGPSYNVELGRRDSLTASQRAANRSIPAPTLNFTGLLSNFQSHGLSMQDLAALSGGHTLGFARCTSFRSRLYNETATLDSSLAESLKSRCPLNGGDDNLAGLDGSPTTFDTFYYDGLLQKKGLLHSDQQLYKGDGSGSDGLVRFYADNPVAFWQAFAASMLKMGSMKPLTGKKGEIRVNCRKVN*

>AcPRX16

MASKAILGLFTLALVLLLVRNSSAQLSTNYYSSSCPNLFSTVKPVLQSAISSEKRMGASILRLFFHDCFVNGCDGSVLLDDTATFTGEKNSNANKNSARGFDVIDNIKTAVEQACPGVVSCADILAIAARDSVVILGGPNWDVKLGRRDARTASLSAANNNIPQPTSDLSTLISMFSAQGLSANEMVALVGAHTIGQARCTSFRARIYNETDIDSSFAKTRQSNCPSTSGSGDNNLAPLDLQTPTTFDNNYYENLIYLKGLLHSDQQLYSNGSTDSLVKTYSMNPTQFCSDFVAAIIKMGDITPLTGSQGEIRKNCRKIN*

>AcPRX17

MKTYPFSIQNSCPSAEAIVKNITWSQVSSNPALPAKLLRLFFHDCFVKGCDASILLDNPQSEKQAGPNQSLGGFVLIDAIKAQLEKQCPGKVSCADIIALFKASLWQVETGRRDGTVSLASNVILPSPLSGFDGLKQSFASKGLSVSDLVALSGAHTMGRANCAFVTPRLFTFNGNGGVDPLLDAAYANSLKKTCPRAPPAAATVEMDPGGSLKFDASYFVALKKNQGLFLSDAALLQDPVAAKLASGFQDQKTFLAAFVQSVRRMGAVGALTGNAGQIRKQCRAVNS*

>AcPRX18

MRRANYKPIFASFLLSVVTIQCMLQSSASLSVGYYKSKCPNAEGIVADAVRSAITGSGDRGIGAGLIRLHFHDCFVRGCDASVLLDGPNTEKTAPPNLSLRGFEVIDTAKSRLESACPGKVSCADILAFAARDLVVLLGGVNYTVPSGRLDGSTSSSSEAMQFLPPPFGDVSQIQSMFAVKGLTLDDMVILSGAHAVGRSHCSSFSNRLYSWNSPDGVDPSMDKNFAAYLKTKCPENGGGDPVVVFNTRYYKLVKAHKVLFTSDQTLLNNAQAAALVNQSSNQPYTWPRSFNKAMVKMGAIEILTSANGGRIRKNCRVAN*

>AcPRX19

MRRRSSNIVAFSCMFFILALQAILPISSASLQFGFYDYNYKCKRTNVELLVRGVVAKAIRKDPGVGAGLIRMYFHDCFVRGCDASVLLDPTTANPTPEKTSVANFPSLRGFEVIDQAKAKLERRCPGVVSCADIVAFAARDAAFFLGRISYPIPSGRLDGRISLQSEVLQNLPAPSFSLSQNEAFFANKGMSPEEMVTLSGAHSIGRSHCSSFTDRLYPTEDPTIDRSFAGILKGMCPQNAAVDGVVPQDIVTPNRLSNQYYKNVKAHKVLFTTDQNLMSSSDTAAMVKENARNQTAWKAKFAAAMVKMGNLVELTGPPGEIRKNCRVVN*

>AcPRX20

MRRRSSNIVAFSCMFFILALQAILPISSASLQYGFYDYDYKCKRTNVELLVRGVVAKAIRKDPGVGAGLIRMYFHDCFVRGCDASVLLDPTTANPTPEKTSGANFPSLRGFEVIDQAKAKLERRCPGVVSCADIVAFAARDAALFLGRISYPIPSGRLDGRVSLQSEVLQNLPAPFFSLSQNEAFFANKGMSPEEMVTLSGAHSIGRSHCSSFTNRLYPTEDPTIDRSFAGILKGVCPQNAAVDGVVPQDIVTPNRLSNQYYKNVKAHKVLFTTDQNLMSSSDTAAMVKENARNQTAWKAKFAAAMVKMGNLVELTGPPGEIRKNCRVVN*

>AcPRX21

MRRANYKPIFASFLLSVVTIQCMLQSSASLSVGYYKSKCPNAEGIVADAVRSAITGSGDRGIGAGLIRLHFHDCFVRGCDASVLLDGPNTEKTAPPNLSLRGFEVIDTAKSQLESACPGKVSCADILAFAARDLVVLLGGVNYSVPSGRLDGSTSSSSEAMQFLPPPFGDVSQIQSMFAVKGLTLDDMVILSGAHAVGRSHCSSFSNRLYSWNSPDGVDPSMDKNFAAYLKTKCPKNGGGDPVVDMNVVTPNKFNTRYYKLVKAHKVLFTSDQTLLNNAQAAALVNQSSNQPYTWPRSFNKAMLKMGAIEILTSANGGRIRKNCRVAN*

>AcPRX22

MATTFSSTTLLALALALFTLLTSAAHGTLSPSFYNASCPKLQTVVRAAMVNVVRKEPRMAASILRLFFHDCFPNGCDGSILLDETPKFLSEKNAPPNFNSVRGFDVIDAIKSKVEKVCRGTVSCADIVALATRDGVNLLGGPSWTVELGRRDSLTANMKAANEFLPGPSSDLAQLLDTFNRRGFTARDVTALSGAHTIGHAHCGFFADRMMGDASIDSAFASLLQQNCSLSAGGGAAEALAPLDRTPKRFDNAYYRDLIAHRVLFHSDQELFNNGPQDSLVRQYSADGTLFAADFAAAMVKMGRLNPLTMATTFSSTTLLALALALFTLLTSAAHGTLSPSFYNASCPKLQTVVRAAMVDVVRKEPRMAASILRLFFHDCFPNGCDGSILLDETPKFLSEKNSPPNFNSVRGFDVIDAIKSKVEKVCRATVSCADIVALATRDGVNLVCTRSTQNTLELGRRDSLTANMKAANEFLPGPSSDLAQLLDTFNRRGFTARDVTALSGAHTIGHAHCGFFADRMIGDTSIDSAFASLLQQNCSRSAGGGATEALAPLDRTPERFDNAYYRDLIAHRVLFHSDQELFNNGPQDSLVRQYSADGTFFAADFAAAMVKMGRMNPLTGTSGQISHGHHLLLHHPPRPHALHPHLCRSRHAIAQLLQRFLPEATDGRARGDGRCRAKGAENGRGCDGSILLDETPKFLSEKNAPPNFNSVRGFDVIDAIKSKVEKVCRGTVSCADIVALATRDGVNLAANEFLPGPSSDLAQLLDTFNRRGFTARDVTALSGAHTIGHAHCGFFADRMIGDTSIDSAFASLLQQNCSRSAGGGATEALAPLDRTPERFDNAYYRDLIAHRVLFHSDQELFNNGPQDSLVRQYSADGTFFAADFAAAMVKMGRMNPLTGTSGQIRLKTSSQLGAPSPKEINPEAQGNSFGPWGFGPCWPGWHDPNGRAVSSHGPAFGPVRIRPVSGRVGPRASLRPSTNQARFGQGCGAQEGRPSTTRRVRPERYRSRVGPRSGPQSC*

>AcPRX23

MLGAHHHHHHHHTFFSLLIFSLLLLMITDQVVVRFAVGDDSGSSSPSPLQAKCGEEFAKLTNCLDYATAKADAPSGECCSAVGAMRKADPACLCYVIQQTHSGTSSAKSLGLQFGRLLALPTACKLVNSSITDCPRLLNLTPSSPDYAIFTNPSKATPTSSSSSSDTTSEEKFVHCVGVIDDSAGRKRPSYGELLPIDVPECGGDRERLSEEDQPNRRHDPRHSSPLLPYCFVEGCDASVMIASPRNDAEKDAPDNLSLAGDGFDTVIKAKAAVEAQCPGVVSCADILTIAARDVVALSGGPNFTVELGRRDGLVSLASRVAGKLPGPDFNLDSLTTLFGKNNLTLFDMITLSGAHTVGFAHCSRFTNRLYRFGSSSSVDPTINPDYARELMQACPANVGPTIAVNMDPVTPITFDNVYYANLAAGLGLFTSDEVLYADGRSQPVVKGFAGDQSSFFAAFAKSMVKLGRVGVKTGRRGQIRRDCTAFN*

>AcPRX24

MKSAYYVVEFLVLVEVILRGGEALSMNYYGMSCPFVEPIVRIVVNQALHKDPTLAAGLLRLHFHDCFVQGCDASVLLDSTSGNTAEKDSPVNLSLRGYEVIDQAKKFLEMQCPGIVSCADIVALAARDAVFWAGGPYYDIVKGRKDGTRSKQEDTFTSLPAPTLNTTGLINVFSQHGFTVWELVALSGGHTLGVARCSSFKSRLSSFDSTHDVDPSMDPTFAKTLLNTCSAGDSAAQPFDATTNSFDNLYFSALQKGDGVLFSDQTLYVSPQTQQIVNFFAMNQAIFFFNFQQGMMKMGSLDVKEGSQGEVRANCRKIN*

>AcPRX25

MFHPLPSSLLLLLLLYALFLSVVYAAEPSSAASAAHLRPGYYSSTCPGAESTVRGVIRRAMARERRSAASVMRLQFHDCFVNGCDASLLLDDTPTMLGEKLSLSNINSLRSYDVIDEVKEEVERECPGVVSCADIIVMAARDAVVLSGGPYWDVKLGRVDSLTASQEDADRIMPSPRANATSLINLFAQFNLTVTDLVALSGSHSIGEGRCFSIVFRLYNQSGSGRPDPHMDSEFRGKLDTLCPKGGDGNVTGGLDATPVVFDNQYFKDLVRLRGFLNSDQTLYSDNEKTREPVECFSRDQEAFFAAFVEGMVRMGDLQSGRRGEIRRNCRVVNGQSMSVDFMDINGQGRRILEE*

>AcPRX26

MGRPNSLAFVASVVAAAIVFLCSSAPADAKLKTTYYERTCPRLQEIVSRVVTDKQITNPTTAAGTLRLFFHDCFVGGCDASVLMATNAFHRSERDADINLSLPGDAFDAVVRAKTNLELECPGVVSCADILALATRDLVSMVGGPFYPVRLGRKDSVVSDPGAVEANLPRANTSMSQIISLFASKRFTVQEMVALTGAHTLGFAHCKEFAERIYNYNGGGSAAFDPSMNPRYAQALQKACANYMKDSTMAAFNDITTPGKFDNAYFQNLGRGLGLLASDHALLADERTRPFVELYAANQSAFFADFAHAMEKLSVYGVKTGRKGEVRRRCDLPNNSL*

>AcPRX27

MISYSRASVLVLAMVLGAFVGPLAAQDPSQLTLDYYAKNCPHAQEIIRAEVECAVRTNPRNAALLLRLHFHDCFVQGCDGSVLLDDTATLIGEKQADQNVNSLKGFDLVDKIKQKLEAECPGVVSCADLLAIGARDGTVLLGGPYWDVPVGRLDSKTASLDLANSDIPAPSQGLITLIAKFWSKGLSATDMVALVGSHTIGVARCFNIKDRIYGDFELTAKFNPSSELYLSKLKETCPAHGGDDNTSPMDYVSPYVFDNAYFEGLVQGEGVLYSDQQMYSSLVGYQTADLVKQYWADPLLFFKHFSDSMVKMGNITNPEGGEVRKNCRFVNS*

>AcPRX28

MGIKKYRMLVLLFSVLLSLLIVETPTAAAQLSQNYYAGICPNVESVVRGVVSQRLQQSFAAGAGTLRLFFHDCFVRGCDASVLIANPNGDDEGHSGADLSLSADAIDLINKAKAAVDGDPQCTNRVSCADILAMAARDVVSLVGGPFYPVELGRLDGKVSTRAIVKYSLPGPTFNLDQLNSLFAANGLTQTDMIALSGAHTIGVSHCDKFIRRLYQFRGPQEFTPRMNLQFLRQMRQVCPLKYSPSTVAMLDASTPFKFDNAFYQNLQQLEGLLASDQILFADRRSRGTVNWFASNQTAFFDAFVSAMTKLGRVGVKTGDDGELRRVCTTVN*

>AcPRX29

MAKIGVVVLLLFLSLYVFPQLGSAQLQQNYYANICPNVETIVRNAVTKKIQETFVTVGATIRLFFHDCFVQGCDASVIIVSTGNNTAEKDNPINLSLAGDGFDTVIRAKAAVDAVPQCTNKVSCADILAMATRDVIALAGGPSYAVELGRLDGLSSTASSVNGRLPPPSFNLDQLTSLFAANGLSQSDMIALSAAHTVGFAHCSTFANRLYNFSPGSPVDPSFNQAYAAQLQAACPPNADPNLAVANDPVTPRVFDNQYYKNLQQGMGLFTSDQVLFTDSRSAPIVNSWAQSPQAFQQAFVTAITKMGRVGVKTGSQGNIRRNCAFFN*

>AcPRX30

MAMAGLSSSSSEMDLDRPILEDYISTDSIQRIPQTLHLRELLDISPTLTEAAGAIIDDSFTRCFKSNPPEPWNWNVYLFPLWCLGVVVRYGILFPLRLAVFIAGWIVFFLTFFPVHFLLPSSNKWRHKMERILVEMMCSVFVASWTGVIKYHGPQPSMRPHQVFVANHTSMIDFIILEQTTAFAVIMQKHPGWVGFIQTAILESLGSIWFNRTESKDREVVARKLREHVQGPDNNPLLIFPEGTCVNNQYTVMFKKGAFELGCAVCPVAIKYNKIFVDAFWNSKKQSFSMHLFRLMTSWAVVCDVWFLEPQYLKPGETPIEFAERVRDMISVRAGLKKVHFYSESCPRVEIIVQQVVQRHFQQDPSVPAGLLRLHFHDCFVRGCDGSVLIDSTDDNIAEKEAPPNLTLRTYDVIDEINTELEKECSGVVSCADMLALAARDGVALAGGPAYALPTGRRDGTVSTLADAHLPSPSFSVDQALAAFQSVNLDLVDLTTLLGAHSMGLCHCGFFIDRLYDYKGSGSPDPTMEPNVLNTLQQKCPPHLVTIDNITEDSIVFMNQVSSTPFHFDNSLYHASLNEKAVLQIDQELAFTDVTSKLAAQYANNPGTFLNQFAKSMIKLGSIGVLTGQEGEIRLNCRVVNNATSNSSTANFGPEN*

>AcPRX31

MVSKAFFVAFSLWLLVLLLFNPSQAHVLFGFYNVTCPNAETMVRDEVGKVLAKQPDLAGALIRLHFVDCFVGSHGPFYTIPTGRRDGNGSVASDVAKYVPSPAATIDDIKAFFAKKNLTVKDLVVLSGAHTLGKAHCYSFSDRLYNFKGNGSSDPSLDANYTMALQNQCKPNDMTTLVDLDPDNATMFDLDYYKLVSNMKGLFASDVALLSDVDAKAYVTRQANATKTDEFFNDFATSMVAMGRLSVLTHQNGEIRKICSKLPTNLLDIYIISFLSGGHAIGRSHCDAFSNRLYNFTGKGDMDPSLDPSYAAQLKKKCKPGDMTTVQDMVPGSATTFDINYFKLVVEKKGLFQSDDALLRNKETRDYVYSHVKVDAESSFFADFGVSMVRMGRVGVLTGKQGEIRKHDFKQCSMERKLRSVIGAVHGNERNNLLSFSGADLELGFYEITCPNAEKIIFEYVKEHISKTPSLAAPLLRMHFHDCFVRGCDASVLIDSTSKNKAEKAAFPNQSLRGFDFVDRVKSLLEKECPGVVSCADIIALIARDSVLVTGGPFWNVPTGRRDGLISNSTEASNNLPAPTFNFTSLQRSFASKGLNLKDLVVLSGAHTIGVSHCTSFDKRLYNFTGKGDQDPSLDKFYAWNLKKNKCKFLNDTKTIVEMDPGSHRTFDLGYYKHLMQRRGLFRSDEALMTNPVTKSYITQILSSPLEEFFKEFALSMEKMGRIEVKTGSFGEIRTNCAVRNS*

>AcPRX32

MASKALLALFLPLALVFLSCLAEAQLKLGFYEETCPHVEDIVREEMTEILKVAPSLAGPLLRMHFHDCFVRGCDGSVLLDSTKGNVAEKDAPPNKSLRGFGSIERVKAKVEKACPNTVSCADILALIARDAVWLSKGPSWPVWLGRRDGRESHAAETKQLPPPKASLPQLIAMFAVKGLDLKDLVVLSGGHTLGTSHCNSFSDRLYNFTGKDNLYDVDPALDKQYITKLRNRCSLTDNTTLVEMDPGSYKTFDTSYYNYVKKRRGLFHSDAALLANDFTKAYVERHASGLYAAEFFQDYSDSIIKMGNVEVLTGYDQGEIRKKCYIVN*

>AcPRX33

MAMRCGKVLLATILCLFLGASSAQGELVEAICNTLAQVIVKDEVTKAFNANSTVAPALLRLHFHDCFVRGCDGSVLLDSTPDNTAEKDAPPNKTLRGFEIIDAIKQRLENSTICAGKVSCADILTYASRDSVVLAGGPFWPVTAGRKDGIISRANETSALPSPRLNLTGLLANFAANNLTEDDLIALSGAHTIGHAHCGSFSNRLYSFNATTPQDPSLNSTYAGQLKESCPVGNLNSTVPMDPVTPDKFDTTYYSDVLLNEGLFQSDAALLSTPSTADKVRLYAGNTTAFNDDFVNSIIKMGAIAPPAGSYGEVRVNCRVSVGREIKRDYKEMAFKGEVDLVLMLMLIAVICFSFLPTPQAQLQVGFYDTLCPAAELIVRDEVQKAMNISPGAASGLLRLHFHDCFVRGCDASVLLDSTANNTAEKDAPPNTSLRGFEVVDSAKSRLEEACFGIVSCADILAFAARDSSVLSGGQSYPVPAGRRDGRVSVSQETFGNLPPPSSDLNQLTQIFANKGFTQGELVTLSGAHTVGGSHCSSFSNRIYNFSVTAGEDPTLDPTYVTQLQQQCPQGGSNASLLVPIDPISPNTFDSSYYTALLANRSLFSSDQALLDSDATSGQVILYANDTDLFLSDFAAAMVKMGQLDVLTGTNGEIRSNCRVTN*

>AcPRX34

MEHLMRAATVVALLVLSLGTACHGALQYGFYSGKCGFKDVEAIIQGVVSARFARDPTIVAALLRLQFHDCFVLGCDASILLDGPSSEKTAFPNLSVRGYDLIDEIKAAVEAACPGVVSCADIIIAAVRDAVALAGGMPYQVPMGRRDGTVGLASNVVLPSPFMSVQQALQIYQQKGFNVFDMVLQLGAHTVGVTHCSFVDGRLYDFNGSGKPDPSMNTALLNDLKQICPPQSSTDNIVDLDQNPTSVNRVDNSYYRQLMQNRGVLPIDQALASDPSTRWIVQLLASTNLFPPLFNNALVKLGSNQGCDASLLLDGPTSEKTAFPNLTVFGYDLIDQIKAALESACPGVVSCADIIIAATRDAVVLAGGQKYEVQLGRRDGTVSQASLVNLPAPEISVAAAIQFYAQRGFGTRDMVLLLGAHTVGVTHCSIVNSRLYNWNGSGKPDPNMDPFYVMVLQNVACPIGQTFDNFVFLDDPSSVLKVDNSYYNQLLRKRGILPIDQALALDPTTKPIVQQLAGNNGLFLTQFGQALVKMGAFQVLTGTQGQIRKNCRRVN*

>AcPRX35

MRKFALVLLLILAISNLTLFPRWGSALQQNYYANICPNVESIVRGAVTQKFQETFNAVGATIRLFFHDCFVEGCDGSVLISSTPTNTAERDYPDNQSLNEDGFDTVDRAKAAVDADPQCTNKVSCADILALATRDLIALAGGPSYAVELGRFDGLSSTVGSVVGKLPAPTFDLDQLTGIFGSNGLSMLDMIVLSACHTVGFAHCGIFSDRLYNFAPGSPMDPTLNKNYAAQLISSCPPNPNPSWHVFLDPVTPGAFDNQYFKNLQQGMGLLFSDQVLFNDTRSQSTVNSLASSSSRFEQAFVVAITNMGRVGVKTGLQGNIRENCRVFN*

>AcPRX36

MGLSSSLSIAIFLLYFTSVLAGPDKKKPDYLTQIDGLDTDYYRKTCPKAEEIISKKVKHWISKDYTLGASLLRLHFHDCAIRGCDGSVLVNTTGSERYAKASKTLRGFDVIDDIKAELEDKCPKTVSCADILTAVARDATHEIGGPYWSLKYGRKDGRKSLAEEAEGVPMGRESVTGLIQFFQSKGLTLLDLVVLSGAHTIGRCSCGSIQYRIYNYTEYGLADPSIDLKFLNFLQRKCRQASEYTELDAVTPTIFDSVYYKNLQQRMGLLSTDQMLYADSRTGPLVKALAAQHNLFYQQFGVSMKKLSKVQVLTGDEGEVRTKCFAFN*

>AcPRX37

MAAEALSILALLLAACMLTAALSLQLNFYQNSCPRAEAIVQQVVQQHFQQNSSVPAGLLRLHFHDCFIRGCDGSVLIDSTDDSIAEKEAPPNLTLRTFDVIDDIKAELEKECPGVVSCADALALAARDGVALAGGDFYDLPTGRRDGTVSNMGDVHIPSPLFSVEQALSAFQSINLDLEDLTTLLGAHSMGFCHCGFFIDRLYDFRGTGVADPRVDPNLLTTLQQKCPPHLVTIENITQDITVFMNQFSTTFHFSNSFYHNVLNEKALLQLDQDLAFTDYTSTLASQYANDAVLFRKRFSESMIKLGSAGVLMGRDGEIRTNCRRVNNATSISSGAKLSAP

>AcPRX38

MAFKRGNNMFLSLALCVLSFTEILSSVSEAQLQVGFYNTTCPSAESIVRDEVTKAVTANAALAAGLLRMHFHDCFVRGCDASVLIDSTTNNTAEKDAPPNKSLRGFEVIDNAKARLEQTCKGIVSCADILAFAARDSIVLTGGYSYKVPAGRKDGRVSVALETLGNLPPPSSDLTQLTQMFANKGFTQDEMITLSGAHTIGYSQCSSFSSRLFSFNSTMSQDPILNSTYASQLKRQCPQGSSNASLSVPIDLTSPNAFDSGYYSNLLANRGLFTSDQMLTSVNTTAVALVRQYANNSALFLSSFATAMVKMGYINVTTGSNGEIRANCRVIN*

>AcPRX39

MRPPAEGAKAMVPQKRPRPGSKSSAALVPDEGLSDAERMILEAIRSKENMGIWTFDLKKLTNLPRGVFDKALRLLQQKGLIKCVVSIHNKSRKIEFEPAKEISGGTWYSHGSLNQEFIGDVRSRCLAQVTRLRIATVEDVWKGIQMSRDSKADFTLQQIKEIVQAMVLDKELEEVKSTGDGDFSVVPAGRPCYRRFERRAPIVGALSAIPCGVCPRIIQAQEGLQKGLYSSSCPKAEDIVRSTVQKFFNNDSSIAPGLLRLHFHDCFVQGCDASVLISGSSSERIAVQNLGLRGFEVVDDAKSQLEAACPGVVSCADILALAARDAVYLTGGPSWSVPLGRRDGRVSSASDATVLPSPTDSLSRGKSAHTIGQTDCLFFRYRLYNFTATGNADQTINGAFLGQLQMLCPEKGNPSKQVALDKDSIAKFDVSYFKNVRDGNAVLESDQRLWGDVVTQNIVHNYAGTIRRLLGLRFDYEFPKSMVKMSSIGVKTGTRGEIRKICSRFN*

>AcPRX40

MDFSKSFLFFAVLSLMSSAADEQLSIAYYATSCPSLHEIVRSVMTQAVAVEPRMGASILRLFFHDCFVHGCDGSVLLDDTPTFRGEKGAKGNANSLRGFKVIDTIKASVEATCKAVVSCADILALAAHDGVQLLGGPCWPVLLGRRDSRKASLSRANKDLPKPNSNLSTLVDAFAAKGLSASAMTVLSGAHTIGQARCTNFRNHIYNDSNITPLFGLFLQQACPRAGSDDFNSPLDFQTPNQFDNAYFQNLMVQTGLLHSDQELFNGGPMDQLVEFYANNAVAFADHFAEAMIEMGNIGPLTGEKGEIRLNCRRVN*

>AcPRX41

MDLRMRRLWFDVNKLKFYHVNAHESHHHMNVFFFFVIFLMISLAMASSSHRFLILCVLSLLALAARAQLSPNFYAKTCPNLQTVVRSAMSQAVAKEARMGASILRLFFHDCFVNGCDGSILLDDTATFTGEKNAGPNANSARGFDVIDTIKTQVEAACKATVSCADILALAARDGVNLLGGPSWTVQLGRRDAQTASQSAANTNLPGPGSSLATLISMFSAKGLTARDMTALSGAHTIGQARCTTFRGHIYNDANVNASFATLRKQTCPQSGGDGNLAPLDIQTPTKFDNAYYQNLVSQRGLLHSDQELFNGGSQDSLVRTYSTSASTFTADFVSAMIKMGNISPLTGTSGEVRLNCRKGCDGSILLDDTAAFTGEQNAFPNKNSIRGLEVIDSIKAQVEASCRATVSCADILALAARDGVVMLGGPTWTVDLGRRDARTASQTAANSDLPGPSSSLSTLVAKFAAKGLDARDMTALSGAHTIGQARCATFRSRIYNDTDIDAGFAALRRQSCPSIGGDDNLAPLDSQTPNRFGNNYYQDLVARFGLLHSDQELFSGGPQDALVRQYSSEESAFARDFATAMAKMATISPLTGTAEEIRLNCRSREFRAMASSQSLFSLCLLSLFACATYAQLSSSFYDHTCPKLPRIVRTAMIQAVRNERRMGASILRLFFHDCFVNGCDGSILLDDTATFTGEKNAFPNRNSVRGFEVIDTIKSNVEAACNATVSCADILALAARDGVFLLGGPFWNVLLGRLDATTASQSTANSDLPGPSSDLSTLISKFAAKGLTARDMTALSGAHTIGQARCATFRTRIYNDTDVDPNFAALRKPNCPASGGDDNLAPLDVQSPNRFGSEPAGAAWSTALGPGALQWGDQLVRLYSFNEGLFAGDFVTAMLKMGNISPLTGSNGQIRLNCRKVN*

>AcPRX42

MALSMSGIVAVLMVLSLAPLSLGGGYLCPEYYDKSCPQVKEIVWSMVAKAVAKEPRMAASLLRLHFHDCFVKVKEIVWSMVAKAVAKEPRMAASLLRLHFHDCFVKGCDASILLDSSGSIVSEKGSKPNKNSARGFEVIDDIKAAVEQACPKTVSCADILALTARYSTVVAGGPNWEVPLGRRDSLGASLSGSNNDIPAPNNTLPTIITKFKRQGLDVVDLVALAGAHTIGFSRCTSFRQRLYNQSGNGLADSTLDESYAMQLRWGCPRSGSDDNLFPLDYVSPAQFDNYYYKNILVGKGLLNSDQILFTKSATTRQLVELYAANIGIFYDHFAKSMIKMGNITPLTGLEGEVRTNCRRINSS*

>AcPRX43

MASHKVLIAISIVSFYFSLSLAFPGFNPFWGGFPVGGVPQWSLYPEYYQFSCPQVDEIVMSVLEREIAKEPRMAASLLRLHFHDCFVQGCDASLLLDDSEKIVSEKGAVPNKNSLRGFEVIDEIKAKVEEVCPGTVSCADIVALAARDSTVLSGGPTWVLPLGRKDSITASLKFANANIPPPNSTLQHLLKLFQRQGLDEVDLVALSGSHTIGMARCVSFKQRLYNQNGDNEADATLEKSFYYTLRSVCPRSGGDNNITPLDFTSPTKFDNYYYNSIMQGKGLLNSDEVLLTGNDAKTMDLVKSYAEDEQLFFEQYAKSIVKMGNISPLTGFNGEVRKNCRSINEDI*

>AcPRX44

MAAASEEKRARRSLGVLGAEREQKKDRGWRRSPTDLGFLGRRREQAGRSLKPSERRKKASPKPAGSRMMRRRRSPASVYPLTSTAGPGGPERLVTPSSRLAIHLHRGLLTGPAHYTLLSHIVEKQSSKKKKKNTKKKKKKKKRMSGVLRWGATLLAVSLLLSLIELGGEGTKKPSNIPPLSDLDDRRIADKPRQLQDDEGEPSTSFSFSSSAPSPQYLEVGFYRRKCPRAEGIVESTVNDLYSRNSSVAPALLRLLFHDCFIHGCDGSVLLDRINGSLSERDAIPNRTLKGFDAIDAIKSAVEAVCPATVSCADILVLAARDGLKLVGGPSYTVLTGRRDSVRSFYDEAQIQIPTPNDTYAKTLSSFASRGFTERETVALLGAHSIGKVHCRFFRDRLYDFAGTGEPDGSLDREMVEEMRAICGGDAAAAAAEEAGMEMGYCREGREVGFGSHYYWRLLEGRGILRADQQLTAGRTVRWVRAYAAGWEGERAFRADFAASQGQVRTRCSRILGS*

>AcPRX45

MATLKNFLVMFIFVIVLSSISLASATNGGYLYPQFYDRTCPKAQEIVRSIVAKAHAQDPRMAASLLRLHFHDCFVKGCDASILLDNSGMIISEKRSNPNKNSVRGFEVVDEIKYALERECPETVSCADILALAARDSTVLTGGPSWEVPLGRRDSLGASLSGSNNNIPAPNNTLPTIITKFKLQHLDLVDLVALSGGHTIGDARCTSFRQRLYNQSGNGLPDATLSEGYAAELRARCPRSGGDQTLFFLDPLTPLEFDNLYFKSLLTGHGLLSSDELLVTQSAATRALVEKFAASNKAFFEQFAKSVVKMGNISPLTGPTRGEIRTNCRKINGEHY*

>AcPRX46

MASKYAYSYSLAFFFIATIVSAQLSSTFYDTSCPGAFHIIKYIVNDAVAKEKRMGASLLRLHFHDCFVNASGCDGSVLLDDTPTFTGEKTASPNNNSIRGFDVIDTMKSHVEAICPQVVSCADILAVAARDSVVALGGPSWTVQLGRRDATTASFSAAKSDIPKPTFDLSALISSFSNKGLSTADMIALAGAHTIGQARCISFRNRIYNETNIDSSLATSLQSNCPSSGGDNNLSPLDASTPTVFDNYYYKNLVNNKGLLHSDQQLYDGGSTDSQVTTYSQNMVKFYTDFAAAMVNMGNISPLTGSSGEIRTNCRKTN*

>AcPRX47

MAISSRAFASLLLALLLLGSASAQLSTNFYSSSCPKVFSTVQSVVKAAIQKEKRMGASLLRLFFHDCFVNGCDGSLLLDDTANFTGEKMAKPNNGSVRGFDVVDNIKSAVENVCPGVVSCADILAITSRDSVVILGGPNWNVKLGRRDSTTASLSGANNNIPFPSNSLSTLISKFSAQGLSTKDMVALSGAHTIGQARCTNFRAHVYNDTDINASFAKSRQSNCPQTSGSGDNNLAPLDLQTPTVFDNDYYKNLVVFKGLLHSDQELFNNGSTDSQVQSYSSSSSSFFSDFVTGMINMGDITPLTGSNGEIRKNCRKIN*

>AcPRX48

MYLYIYISLSLSKMMHKYHVHALWFVITLFLVVHQLFSPTVAALEVDYYRLTCPMAEKIVRRTVTRAVDRDRGLAPALIRMHFHDCFVRGCDASILLDPTATNRTEKLSPANTPSLRGYEVVDEAKARLESHCPQTVSCADIIAFAARDSACLAGGPEYAVPAGRRDGAVSRESEVLPNLPLPNSTLDQLIQIFSSKGLAVEHLVALSGAHSVGVSHCSSFAHRLYGFNATHAQDPALDRSFASLLKANCPPSSGTDTARTDPTVPLDSKASDRLDVRYYMNLMAGRGLLGSDQALVSGPVTAALVAVHAAEPWRWKEEFAAAMVRMGSIEVLTGEEGEVRKDCRAVNG*

>AcPRX49

MTLVLTLLACVIHALNLANKSDASNKTCFGGIDGSIRFDIYQTTCPQAEDIIFAGVEEAVANDPRMAASLLRLHFHDCFVDGCDASVLLDDTPNFVGEKTAGPNLNSLRGFEVIDSIKAELELACPETVSCADVLAIVARDSVVLSGGPTWQVEVGRKDSRSANRQAANTNIPAPTSDVATLIGKFQNLGLSSKDMVALSGAHTIGKARCSSFSSRLNGLVGSSDASMGDRDFLQSLQQLCASSNNTLADLDLSTPATFDNQYYVNLISADGLLQSDQALTSAGSGDVAALVEAYAIDPELFFEDFKASMVRMGRLAMSGGGEVRRSCRVVN*

>AcPRX50

MIDLYSSYKHHNFHILNCSVRMASRCLYKLFLPLFLLLLLHPPALSVAQGLQMDFYQYSCPKAEAIIGSEITKVISRAPSLAGPLLRMHFHDCFVNGCDGSVLLNSTKDNSAEKDAIPNVSLRGFGVIDGIKAKLEEACPGVVSCADILAVVARDVVVLRDGPYWDVLTGRRDGRRSVAADALNNLPPPFFSASQNLNQFFLPKGLDAKDQVVLLGAHTIGTSHCSSFSDRLYNFSGTGTSDPTLDKHYLPKLMNKCKPGDTKTLVEMDPGSFRTFDTGYYKQVTKRRGLFTSDETLMLDPFSRHYIERQAYGPATEFFRDFGVSMVKMGSIQVLTGSQGEIRRHCAFVN*

>AcPRX51

MKGGYRCVSYNLFLSTLVVLCFSQSSVRSQLTPNFYAQSCPNVLKVVKARVFAAMKNEFRMGASLLRLHFHDCFVNGCDGSILLDGSDSEKLALPNKNSVRGFDVVDDIKAAVESACSATVSCADILAIAAKEAVVLSGGSSWNVLLGRRDGLAANQSGANSGLPSPFDPIDTIISKFNDVGLNTTDVASLSGAHTIGRARCALFSNRLSTLDPVMAADLQALCQSGDGNTTTALDRNSADVFDNHYFQNLLNNRGLLSSDQGLFSSSEGIAATKSLVETYSNDKCTQEEEEEMSSYSSSISSTTLVIFFVLLLRGTEAQLSSDFYDHTCPDVYHIVRSVIKHAHKSDIRIYASLNRLHFHDCFVQGCDGSLLLDSSSTIVSEKNAIGNNNSLRGFPVVDAIKEALEHACPGVVSCADILALAAEASVELSGGPKWCVLLGRRDSTTANLTGAQNLPSPFDSLQALRSKFSAVGLNDTDLVALSGAHTFGRAQCGIFSYRLYNFSGTGQPDPTLNGDYRVQLQQNCPQNANFNRLNNLEPTMPDIFDYNYFLDIENNRGLLQSDQELLSAVDAVSTTAPIINSFASDQSTFFSNFAASMINMGNISPLTGDQGEIRKNCRVKKNKKKKMLSNYSASLSSVVLTIFAVLLLRGSEAQLSSTFYDRTCPNVSAIVTSVIKQAHQSDVRIYASLTRLFFHDCFVQGCDGSLFLDSTPTIQSEKSAPANNNSARGFPVVDNIKAAIENVCPGVVSCADILALASEASVDLSGGPSWSVLLGRRDSTTANLASAMNLPSPFDTLSKLRSKFSNVGLSDTDLVALSGAHTFGRAQCRFFNQRLYNFNNTGRPDPTLDTTYLATLQQSCPKNANLNGLNNLDPTTPNKFDKNYYSNVENKRGLLQSDQELLSSSDAASTTAPIVNSFASSKSTFFRAFATSMINMGNISPLTGSRGEIRSNCRKINGS*

>AcPRX52

MNHMSPLSSSSSSSVVAVVVAVFFFCFFSSSFVASSVGISNAACRNATRKRAAPVRHALSPNFYGATCPNVEEIVASVAAARYRECPAAGPATIRLFFHDCFVEGCDASILIAPTSGGGATEKVERDVEENKNLAPEAFEVVEIAKAAVESKCPGVVSCADILAIAARDFVHLAGGPYYPVKQGRRDSKVSLAWKVRPNLPRANATVDDLLRLFRLKGLTRRDLVALSGAHTVGFSHCDQFVARLYDFRGSRGPDPAMDPRLLKALRMSCPHAGGNADVVAPFDVRTPFAFDHAYYANLEAKMGLLATDQALFLDARTRPVVQEMGRDKASFFAAFAEGMEKMGEIRVKKGRTGEVRRLCSESLPMI*

>AcPRX53

MMKLAMSFALLASLVTALPPLCLAASANTNGGFLYPQFYDHTCPKAQQIVWSGVAKAHAKDPRMAASLLRLHFHDCFVKGCDASVLLDSSGTFLSEKRSHANLNSARGFEVIDEIKFALEKECPLTVSCADILALAARDSTVDSLGASLSGSNNNIPAPNNTLPTIITKFKLQGLDLVDLVALLGSHTIGDARCTSFRQRLYNQTGNGLPDGTLNAAYAAELRARCPRSGGDQNLFFLDPVSPFKFDNQYYKNIVAGKGLLNSDQILFTRSAATMALVEQYANNNELFFDHFAKSIVKMGNIAPLTGSRGEIREHCRKVNHH*

>AcPRX54

MSSYSSSISSTALVIFFVLLLRGIEAQLSSDFYDDTCPDVYDIVRNVIEQAHESDIRIYASLTRLQFHDCFVQGCDGSLLLDNSSTIVSEKNSPANKNSARGFPVVDAIKAALEDACPGVVSCADIIVLAAEASVELSGGPSWCVLLGRRDSTTANLTSSQNLPSPFDSLQTLRSKFSAVGLNDTDLVALSGAHTFGRAHCRFFSGRLYNFSGTGRPDPTLNGAYRVQLQQNCPQNANPNGLNNLDPTTPNIFDYYYFLNVENNRGLLQSDQELLSAAGAASTTAPIVNSFASDQSTFFCNFAASMINMGNISPLTGDQGEIRENCRVVNDS*

>AcPRX55

MASASASSSLSTMSLLLLLAICISSLCSPTRADEFDYPPLVNGLSFSFYKSKCPNLDSIVRDFLKNSFKNNIGLAAGLLRLHFHDCFVQGCDGSVLLDGSASGPGEQQAPPNLTLRPAAFKAINDLRALIDKACGQVVSCADVVALAARDSVYLSGGPKYKVPLGRRDGLTFATRNVTLASLPAPTSNVTKLLSVLSNINLDTTDLVALSGGHTIGLAHCTSFTNRLYPTQDPVMDKTFAKNLELTCPASNTTNTTVNDIRSPNTFDNKYYVDLLNRQGLFTSDQDMYTDKRTKSIVTSFAVNQSLFFKQFVYSIVKMGQLSVLTGGKGEIRANCSARNGDNKFLWSVVGGEGGESASVF*

>AcPRX56

MASASASSSLSTMSLLLLLAICIPSLCSPTRADEFVYPPIVNGLSFSFYKSKCPNLESIVLGFLKKSFKNDIGLAAGLLRLHFHDCFVQGCDGSILLDGSASGPGEQQAPPNLTLRPAAFKAINDLRALLDKACGQVVSCADVVALAARDSVFLSGGPKYKLPLGRRDGLTFATRDVTLASLPAPTSNVTKLLSVFSKINLDATDLVSLSGGHTIGLAHCTSFTNRLYPTQDPVMDKTFAKNLKLTCPASNTVNTTVNDICTPNKFDNKYYVDLLNRKGLFTSDQDLFTDQRTKSTVTRFAVSQPLFFEKFVFSIVKMGQLSVLTGGEGEIRANCSARNGDNKFLWSVVGGEEGESARVF*

>AcPRX57

MAISTTFRSIFWPYILAMALTPQWVSLAQLQVGFYSNSCPNAESIVSSVVREFAQSDVTILAALLRLHYHDCIVQGCDGSVLIQQPAGAGNLEKSAQNHAGLRGFEVIDRAKEQLEAQCPGVVSCADILALAARDSVFLSRGPFYELPTGRRDGRVSNVSDADNMPEVHESIDVLKAKFAAKGLSGNDLVLLSAAHTIGTTACFFLESRLYNFGGRGGSDPSINPSFLPELESQCPQGGDVNVRLPLDRGSETVFDAQILRNIRSGFAAIESDAQLYADPSTRAIVDSYLDPLSPLFGPFFRSDFADSMVRMGRIGVRTGVDGEVRRNCSAFN*

>AcPRX58

MASRASALSCCCCCFFILTSLASAQLSSTFYDSSCPLALLTIETAVVVAVTAERRMGASLLRLHFHDCFGCDGSVLLDDTSTFTGEKSAFPNKGSLRGFDVVDTIKALLEIVCPQTVSCADILAVAARDSVVALGGPSWTVLLGRRDSTTASLSAANSDLPSPFSSLNGLISAFAKKGLSTSDMVALSGAHTIGDARCTNFRTRIYNETNIDSSFATALQANCPFSGGDNNLAPLDTSTPTIFDNFYYNNLVNKEGLLHSDQQLFNGGSTDSQVSSYTTNSAQFFRDFATAIGCDGSVLLDDTPTFTGEKSAIPNRGSLRGFDVVDRIKAQLEIVCPQTVSCADILAVVARDSVVALGGPAWTVLLGRRDSTTASLSTANSDLPSPFSNLNDLISAFAKKGLSTSDMVALSGAHTIGDARCTNFRTRIYNETNIDSSFATALQANCPFSGGDNNLAPLDTSTPTIFDNFYYNNLVNKEGLLHSDQQLFNGGSTDSQVSSYTTNSAQFFRDFTTAIVKMGNISPLTGSSGEIRTNFCVLSSTFYDTSCPQALPTIKKAVIAAVAKERRMGASLLRLHFHDCFGCDGSVLLDDTASFTGEKTAKPNANSLRGFDVVDTIKSQIESVCKQTVSCADILAVAARDSVVALGGPTWAVLLGRRDSTTASLSEANSDLPCVSFRDRIYNDTNIDPTCVTVLKAKCPQSGGDDNLAPLDAVTPTIFDNFYYKNLLNKKGLLHSDQQLFNGGSTDSQVSSYATNFAKFFSDFPSAMINMGNISPLTGTDGEIRTN

>AcPRX59

MASVLSLLSCVVLVLAASFNPTRGQLDPCFYDKLCPHALPAIKAVIEETIAVEPRMGASLLRLHFHDCFVNGCDGSNLLDDTPFFTGEKNAAPNKNSARGFEVVDRIKDAVNYACGGNVVSCADILAVAARDSVAALGGPSYQVQLGRRDSTTASAAAANSSIPAPTFDFDRLLANFNSHGLSLVDLVALAGGHTLGFARCINFRDRLYNETATLDSGLASYLKSQCPLSGGDDNLAPLDDTPVRFDTSYFDRLLRRKGLLHSDQQLFKGDWGGADGLVSFYSSNPEAFAKDFGESMIRMGALSPLTGSAGEIRLNCRRSFTLFALIPAIFFPAFAQLTSDFYDSICPQALSTIQATVEAAIIQEPRMGASLVRLHFHDCFVNGCDGSILLDDTPAMTGEKTAKPNKNSARGFDVVDQIKSAVNDACLGNVVSCADILAVAARDSIVALGGASYDVLLGRRDATDASLAAANANIPAPTLDLAGLVSNFQSQGLSLSDLVVLSGAHTLGFARCVSFRNRIYNETADINSDFAASLQAQCPPDAGDGDDVLASLDDTPFVVDTDYYQGLLQNQGLLHSDQQLFQGDGSDSDNLVQFYSQSPTDFWSDFGTAMINMGNLSPLTGDDGEIRENCRVVNQD*

>AcPRX60

MGSSPFLSLFLFFFFLALVSLTPLAIGGGDEVGVKSTGLVLNFYKESCPQAEEIISQQVKLLYKRHKNTAFSWLRNIFHDCAVQSCDASLLLDSTRRSLSEKEADRSFGMRNFRYIETIKEAVERECPGVVSCSDILVLSARDGIAELGGPYIPLRTGRRDGRKSRGADLLEEYLPDHNESISSVMDKFHALGIDTPGVVALLGAHSVGRTHCVKLVHRLYPEVDPALNPDHVPHMLKKCPDAIPDPKAVQYVRNDRGTPMKLDNNYYRNILDDKGLLQVDHDIAHDKRTRPFVKKMAKNQNYFFREFSRAITILSENNPLTGDEGEIRKRCNAVNKRH*

>AcPRX61

MRRRLSYLICVMCVVLGFVACAEAEAELKLGFYDKTCPKAETIIADYVKKHIPNAPTVAATLLRTHFHDCFVRGCDASILINSTKNNEAEKDSAPDVTLRGYDFIDRVKSLVEEECPGVVSCADIIALVARDAVGVIGGPYWEVPTGRRDGRISNKTEALNEIPGPQFNFSRLKASFASKGLDLKDLAVLSGAHTIGIAHCNSFNKRLYNFSGKGDNVLDEDPSLDPFYAANLRKNKCKTPTDNTSIAEMDPGSFLTFDIDYYRNLLKRRGLFQSDAALLTNSVTKSYVVELLDGPLETFFKEFAVSMVKMGKIQVKTGTEGEIRKNCAVINS*

>AcPRX62

MPHPLSVPLPLPLLMILTLTLTLTLGSGSGAPRMKVRVKLRASYYRRTCPGAERIVAEAVTAKQIANPSTAAGALRVFFHDCFAGGCDASLLISPDSGPGSGSSAAAERDAEINRSLPGDAFDAVARAKAALEQACPGAVSCADVLALAARHLVSMLGGPFYPLRLGRRDALFSPAPSSAHVAPDLPRTNMTAPQLIALFASKGFTVQEMVALAGAHTVGFSHCKEFAGRIYNFENRGPNAFDPALNPRFAQGLQRACASYLKDPTISTFNDVMTPGKFDNLYFQNLPRGLGLLASDAALFVDTRTRPFVERYAANQTAFFEDFALAMEKLSAYGVKTGRDGEVRRRCDAFNDHP*

>AcPRX63

MGLKLMSLLCKANILLCALFVEALVSSAAVSDSSNSLDLSSDFYSLSCPNVELLVKETVSSASSFDPTLPGKLLRLLFHDCVVEGCDGSVLIQGNGTERSDPANKSLGGFEVVESAKRLLEVLCPATVSCADILVLAARDAVEIAGGPPVRVPLGRKDGLNSSASNVRPNMVDTSFSLDEMVRLFSSKGLSLDDLVILSGAHTIGLAHCNAFSDRFKEDSKGNLVPIDSSLEESYALDLAKKCKAGASSSVTVSNDPVTASFFDNQYYKNLLDGKGLFQSDSVLISDSRTKGKVETFSENQDSFFDGWGDSFVRLSSVGVKTGDEGEIRVSCSSMNG*

>AcPRX64

MRRRSSNIVAFSCMLFILALQAILPISSASLQYGFYDYDYKCKRTNVELLVRGVVAKAIRKDPGVGAGLIRMYFHDCFVRTFGAIEFSAVKSKILIFSIR*

>AcPRX65

MAALIASFLLAFSLLLVGVNALMVDYYAETCPGVEEAVEEAVKKATANDNTVPAALLRMHFHDCFIRGCDGSVLVDSQGNNTAEKDGPPNISLHAFYVIDNAKKAVEELCPGVVSCADILALAARDAVAISGGPIWEVPTGRKDGKISRASDTSQLPAPTFNFSQLKQSFSQRGLSTGNLVALSGGHTLGFAHCSSFQNRIHNFDATDDVDPSMNPAFADSLRKACPAHNKVRSAGSAMDSTNTVFDNTYYKLLLQGKGLFSSDEALLTHRKTMELVARFADSQKAFERAFVKAMIRMSSLTGGDGVRLDCRVVT*

>AcPRX66

MANPPWSSPFPSSAAAPMAAAAAAGERRVLHCDGCGASEFDGGDDGFYYCRHCGSQSQDVVDTGCADEDVVGDGSGRGALYSFFHHRSRPSAAAATPSAPKLSREDILRSLARSVASLKSGDEEEEERGGKGEGERPYAFEEEEGEEPRDFGGAHRERERERERERGAGPGLGADAEAAGAAIRARYVQGLQVMLQRQCEALVEQFGVGPLICGLAGTIWLRYVAASQVLDKGWARKVITESKAAAAATPSRDDKQEDRRKPSRHIRAKYRSEPHDSYGRRVIYIFFRALRKTIPIYSSLAICFLVCHIAREAVLPTDIYQWAIEGKLPYLTAFVELDKYLGTPSSDCPLSTRFLFRPIRVVGPWQLEETAASISQAIGLRLPSVNFYAIARRYLHELSLPVEKILQHACRLYEWSLPAELWLSSNASRLPTRVCVMSILIVTLRILYNIHGQGTWEMGLSDIADSLSCGHQFEENVDESNSSSRQKLGVADQGSRACQSTTNSSTVKNLMDNKTSEFETKELLSILEAAYGKINVRHDYAKDLPSYLKYCKDVAFTGITTSYDEETLIERLWDIYSKKEDDNQQDDVKAEFSCLKGKRSSNEAPATEFFDLKRPHRNSSGRFTCSKSFAIERMKQEMEENGFHYLPPRAQKRTEGYLHYKRKKVDGRLIYVAHADYYILLRSFAKLAQVDVRIMHLGVLKLERRLGWIELRIDSSLNALPDLPVYPMAKFLLIFLLLAILSLAGADGVLSPYYYLESCPLAEEIVRHTVEGALYQDPRIAAGLLRLHFHDCFVLGCDASVLLDDADGIVSEKNAVPNKNSLRGFEVIDFIKALLEEACPLTVSCADILAIAARDAVHLRGGPTWDVYLGRKDSLKAASISTTNQMIPAPNSSLEMLISNFKDHGLDIVDLVSLSGSHTIGRSRCVSFKDRIYGLPLNEEYDERRSIFFHILRSICPESGGDDKVVPLDSRTSRRFDNLYYINLLRGEGLLHSDNALASEEDAEESVLSLVWAYAEDQELFFQHYTESIIKMGNINVLTGIEGEVRHNCRFVNSYYH*

>AcPRX67

MSSSYVLLLILLYTFTTSSVGAQAAGVEKGFYKKSCPQAEQTVQSIVWDRVASNGALPAKFLRMFFHDCFVRGCDGSVLIDSTANNTAEKDATPNKSLAGFDVIDDVKTALEKACPQTVSCADIVALAARDSVSFQFKKPIWEVQTGRRDGTTSQASEANANIPSPSSDFTTLVNDFANKGLDVKDLVVLSGAHTIGVGHCNAFSKRLYNFTGKGDADPSLNATYAAFLRTQCKSLSDNTTTVPMDPGSSTDFDNHYFANLKLGQGLFQSDAALLTDGRSTKVVNKLLDAGDFLDAFKNSIRNMGEVGVLTGSSGEIRKNCRVVNS*

>AcPRX68

MSSKGCSFHITTCFAATVLLLLLSTFSCEAQLTPNYYDYACPQALSTIRAAVRAAIAKERRMAASLIRLHFHDCFVQGCDASLLLDDAPNIVSEKNAFQSFKSLRGFEVIDSVKSAVEEVCPGVVSCADILAVVARDSSEYVGGPTWTVKLGRRDSTTANKDLARANLPNATNDLDALVSIFARQGLSVKDMVALSGAHTIGLARCVSFRDHIYNDTDIDAGFASTRRWRCPSAGGDCNLAPLDLVTPYSFDNNYYKNLLQEKGLLHSDQVLFNGGATDDIVRVYSANQAAFYADFAAAMVKMGDICPLTGAAGEIRKVCRLVN*

>AcPRX69

MSTTSKGSGFHIIITSFAATVLLLLLSTFTCEAQLTPNYYDYTCPQALSTIRAAVRTAITKERRMAASLIRLHFHDCFVQGCDASLLLVDAPNIVSEQNALQNFKSARGFEVIDGVKSAVEKVCPGVMSCADILAVVARDSSEYVGGPTWTVKLGRRDSTTANKDLARADLPTAFDDLDALVSSFARQGLSVKDMVALSGAHTIGQAQCVTFRNRIYNETDIDAGFASTRRRRCPSAGGNTNLAPLDLVTPNSFDNNYYKNLLQQKGLLHSDQVLFNGGATDDIVRGYSTNRAAFYADFAAAMVKMGDISPLAGAAGEIRKICGLVN*

>AcPRX70

MSHPCSLLLALNLITITLALLSQRSQAQLSPSFYNSTCSNVSAIVRSTVQQAQTSDVRIYASLTRLFFHDCFVQGCDGSVLLVNSSTIQTEQDAVPNKNSIRGFNVVADIKTAVENACPGVVSCADILALTAEASVNLAGGPSWTVLLGRRDSTTANITAANNLPSPFDNLTTLEQKFAAVGLNDTDLVTLSGAHTFGRGQCQFVTPRLYNFSSTVNTDPTLNSTYATTLEQNCPQGGNASVLNNLDPITPNTFDSNYYINLQNHQGLFTSDQELLSDPNATTTASIVNNFAGNQSAFFDSFVQSMINMGNISPLTGSSGEIRTNCSVVNSGSGLELHIRIQHDSDKGSAAE*

>AcPRX71

MIRMEKRLRLFLLSFILSLLVSPPPKAAAAQLDPNYYANICPNLENLVRGAVVKMMSQTPVAAPATLRLFFHDCFVMGCDASIMIISSDGSDEWRNPADFSLKAEGFETVLAAKAAVDGDLQCTNKVSCADILALATRDVVSLSGGPTWPVELGRYDGKVSLGSDVVLPSPDFNLDQLNGMFGSFGLTQTDMIALSGGHTIGASSCMFFYNRLYPGDPTVSPGFLLQLQQSCPNPLLDPNAFAFLDAATPLQFDNAYFRNLQQGMGLLNSDQVLFNDPRSQGTVNLFASNQSAFFSAFVDAMTRLGRLGTKTAVDGEIRRDCRFVN*

>AcPRX72

PHTQEWYFPNEEKTPEIPNKISPFLPPQTFRALPNLILPEESAAVVVAAAAAVTMNKDVLTVLVFLLVVAIARHATASVELRETASRLRVGYYNSTCPKAEEIVREAVTARHAVDPTIAAGLIRLYFHDCFGCDASILLDETPSGDPVEKSSSANGFTLRGLDVIDAAKSALESACPATVSCADVLAFAARDAAALAGLRRYAVPAGRRDGVVSLASDVPSGLPSPAYSVDNMTALFARKGFSPAEMVVLAGAHSVGGAHCVAFSNRLYNFSATAEQDPAMDATYAAHLRTLCPLPATLADVAKQPKVDFDPRTATTLDTSYYVGLQTGRGLLASDQALMRDRRARAIVQRMAQDPRKWARKFAKAMVKLGEVEVLVGDKEGQIRTDCRFVNADTDIPFFGIIPGGIL

>AcPRX73

MSTSLKSLAQQKCLVLGFALVAVEGGKDGGWLESLGLGLGWKQQQGFGTSLGSSCSSSPREPAALARKPYLRCYSFKVVSIASHSLLYPATLLEVRAEERVKRLRRRDGNLSARTPLSTFGTFPRKQGAHTVGVTHCSFVDGRLYDFNGSGKPDPSMNTALLNDLKQICPPQSSTDNIVDLDQNPTSVNRVDNSYYRQLMQNRGVLPIDQALASDPSTRWIVQLLASTNLFPPLFNNALVKLGSNQVLTGTQGLAQTMFFHTELQTTLHYSCRRREKAKMVSRGAAVIFALLALNLAVSCQASLQYGFYKGKCKNQDVETIIRNIVTASWFGDQKIVAGLLRLQFHDCFVRGCDASLLLDGPTSEKTAFPNLTVFGYDLIDQIKAALESACPGVVSCADIIIAATRDAVVLAGGQKYEVQLGRRDGTVSQASLVNLPAPEISVAAAIQFYAQRGFGTRDMVLLLGVHTVGVTHCSIVNNRLYNWNGSGKPEPNMDPFYVMVLQNVACPIGQTFDNFVFLDDPSSVLKVDNSYYNQLLRKRGILPIDQALALDPTTKPIVQQLAGNNWTLPHPIGQGPGQNGCVQV*

>AcPRX74

MTLVLTLLACVIHALNLANKSDASNKTCFGGIDGSIRFDIYQTTCPQAEDIIFAGVEEAVANDPRMAASLLRLHFHDCFVDGCDASVLLDDTPNFVGEKTAGPNLNSLRGFEVIDSIKAELELACPETVSCADVLAIVARDSVVLSGGPTWQVEVGRKDSRSANRQAANTNIPAPTSDVATLIGKFQNLGLSSKDMVALSGAHTIGKARCSSFSSRLNGLVGSSDASMGDRDFLQSLQQLCASSNNTLADLDLSTPATFDNQYYVNLISADGLLQSDQALTSAGSGDVAALVEAYAIDPELFFEDFKASMVRMGRLAMSGGGEVRRSCRVVN*

>AcPRX75

MATTFSSTTLLALALFTLLTSAAHSTLSPSFYNASCPKLQTVVRAAMVDVVRKEPRMAASILRLFFHDCFPNGCDGSILLDETPKFLSEKNAAPNFNSVRGFDVIDAIKSKVEKVCRATVSCADIVALATRDGVNLLGGPSWTVELGRRDSLTANMKAANEFLPGPSSDLAQLLNTFNRRGFTARDVTALSGAHTIGHAHCGFFADRMMGDTSIDSAFASLLQQNCSLSAGGGAAEALAPLDRTPERFDNAYYRDLIAHRVLFHSDQELFNNGPQDSLVRQYSADGTLFAADFAAAMVKMGRLNPLTGTSGQIRLHCSRVN*

>AcPRX76

MTLVLTLLACVIHALNLANKSDASNKTCFGGIDGSIRFDIYQTTCPQAEDIIFAGVEEAVANDPRMAASLLRLHFHDCFVDGCDASVLLDDTPNFVGEKTAGPNLNSLRGFEVIDSIKAELELACPETVSCADVLAIVARDSVVLSGGPTWQVEVGRKDSRSANRQAANTNIPAPTSDVATLIGKFQNLGLSSKDMVALSGAHTIGKARCSSFSSRLNGLVGSSDASMGDRDFLQSLQQLCASSNNTLADLDLSTPATFDNQYYVNLISADGLLQSDQALTSAGSGDVAALVEAYAIDPELFFEDFKASMVRMGRLAMSGGGEVRRSCRVVN*

>AcPRX77

MVLSLAPLSLGGGYLCPEYYDKSCPQVKEIVWSMVAKAVAKEPRMAASLLRLHFHDCFVKLSREHAVELEKGSKPTRTRRREVSSHRRPLSRGAHTIGFSRCTSFRQRLYNQSGNGLADSTLDESYAMQLRWGCPRSGSDDNLFPLDYVSPAQFDNYYYKNILVGKGLLNSDQILFTKSATTRQLVELYAANIGIFYDHFAKSMIKMGNITPLTGLEGEVRTNCRRINSS*

>AcPRX78

MEQHYLRGLVVGLVLLLALRIAEAGLSPNYYETSCPNVESIVRQAVEKKKNATVVTVPATLRLFFHDCFVEGCDASVLIASPNGDVGRTRRTTSPSAATGVFDTGHQAKEAVEAECPPAWSPGADILAIAPSYVCGSSNTYQYYHSAGGPTQRLNSPARTGPHLHQPSAVAGTLPARLNNAPNTTSLFGKHNLNLARRTSHSRRRPHGRFAHCTRIRPTACTYSARRCLVDPRSSGVRGAAERCARRIRRSHDGGEHGPGHGARYFDSYLLPKPVRRPRAFRSRPSALPRHTGRSPRASDDIAGTSSSSSRAFAQQAMVKRSGGSAQAGKLGPDQERLHHVQLSCTLRCEI*
